# Genetic and serum biomarkers of NSAID hypersensitivity reactions

**DOI:** 10.3389/fphar.2025.1502755

**Published:** 2025-10-02

**Authors:** G. Amo, J. M. García-Menaya, M. Martí, J. Gómez-Tabales, J. A. Cornejo-García, N. Blanca-López, G. Canto, I. Doña, M. Blanca, M. J. Torres, J. A. G. Agúndez, E. García-Martín, P. Ayuso

**Affiliations:** ^1^ University Institute of Molecular Pathology Biomarkers, Universidad de Extremadura, Cáceres, Spain; ^2^ Allergy Service, Badajoz University Hospital, Badajoz, Spain; ^3^ Allergy Research Group, Instituto de Investigación Biomédica de Málaga y Plataforma en Nanomedicina–IBIMA Plataforma Bionand, Málaga, Spain; ^4^ Allergy Unit, Hospital Regional Universitario de Málaga, Málaga, Spain; ^5^ Allergy Service, Infanta Leonor University Hospital, Madrid, Spain; ^6^ Research Consultant, Málaga, Spain

**Keywords:** vitamin D, receptors, IgE, adverse drug reactions, non-steroidal anti-inflammatory agents, polymorphisms, genetic, biomarkers

## Abstract

**Background:**

Hypersensitivity reactions (HRs) to non-steroidal anti-inflammatory drugs (NSAIDs) constitute a significant clinical concern due to their frequency and possible severity. These reactions may involve specific immunological pathways, including activation of the high-affinity IgE receptor, or non-immunological mechanisms that result in similar clinical manifestations. Additionally, vitamin D plays an important role in the regulation of the immune response and therefore may be relevant to the development or progression of HRs to NSAIDs.

**Methods:**

To identify new single nucleotide variants (SNVs) associated with HRs, we analyzed the exonic regions and the 3’UTR of sixteen genes related to the vitamin D pathway and the high-affinity IgE receptor, namely, *FCER1A, MS4A2, FCER1G, VDR, GC, CYP2R1, CYP27A1, CYP27B1, CYP24A1, RXRA, RXRB, RXRG, IL4, IL4R, IL13*, and *IL13RA1* in a cohort of 1,962 participants, including 982 patients with HRs to NSAIDs and 980 controls. Additionally, serum levels of vitamin D and IgE were measured in these participants.

**Results:**

We identified 216 variants located in the exonic and 3′regions of these genes, 186 of which were novel. Multinomial analyses identified associations between patients with single NSAID induced urticaria/angioedema or anaphylaxis (SNIUAA) and SNVs in genes related to vitamin D (*VDR* rs78783628, rs739837, rs731236; *CYP24A1* rs2762934). In contrast, among cross-reactive (CR) NSAIDs HRs patients, none of analyzed SNVs remained statistically significant after adjustment for multiple comparisons. Notably, the *FCER1G* rs11421 variant was associated with an increased risk of anaphylaxis in CR NSAIDs HRs patients. Furthermore, no statistically significant differences in serum IgE levels were observed between SNIUAA and CR NSAIDs HRs patients. However, a haplotype comprising SNVs in the *RXRB* gene was significantly associated with elevated serum IgE level in both patient groups. Conversely, SNIUAA patients exhibited significantly higher mean vitamin D levels compared to CR NSAIDs HRs patients and non atopic controls.

**Conclusion:**

Our study highlights a significant association between SNVs in genes related to the vitamin D pathway, retinoid receptors and the high-affinity IgE receptor with the risk of NSAIDs HRs. Additionally, the correlation between IgE levels and clinical phenotypes suggests a potential role for this biomarker in the pathophysiology and stratification of these reactions.

## 1 Introduction

Non-steroidal anti-inflammatory drugs (NSAIDs) are usually prescribed for treating acute and chronic mild to moderate pain-related disorders associated with inflammation, as well as to reduce fever (Ho et al., 2018). Current studies have shown a rising trend in NSAID consumption in developed countries (Davis et al., 2017; Gómez-Acebo et al., 2018; Baigent et al., 2013). These drugs inhibit the biosynthesis of prostaglandins and thromboxane by the inhibition of the prostaglandin endoperoxidase synthases (PGHS-1 and PGHS-2; also known as COX-1 and COX-2) (Brune and Patrignani, 2015).

Despite NSAIDs' therapeutic benefits and their efficacy, these drugs are frequently involved in adverse drug reactions (ADRs) causing up to 30% of hospital admissions for ADRs (Pirmohamed et al., 2004). NSAIDs are the most frequently involved drugs in hypersensitivity drug reactions (HDRs); their prevalence ranges from 0.5% to 5.7% among the general population, rising to 30% in patients underlying respiratory or cutaneous diseases (Doña et al., 2011; Doña et al., 2012; Szczeklik and Stevenson, 2003). This prevalence also carries an economic burden.

Consequently, the use of alternative drugs to manage hypersensitivity reactions to NSAIDs (HRs) resulted in a 1,142.12% increase in annual costs in 2016 (Cubero et al., 2017). The mean cost of assessing suspected NSAID hypersensitivity in Spain was €239.53 (SD = 140.59) (Sobrino-García et al., 2020). According to the European Academy of Allergy and Clinical Immunology (EAACI), there are five clinical entities, grouped into two main categories based on the underlying mechanism: i) cross-reactive NSAIDs HRs (CRs), and ii) selective NSAIDs HRs (SRs) (Kowalski et al., 2013). The mechanism of CR is non-specifically immunologically mediated and seems to be related to COX inhibition. However, this mechanism is not fully understood. SRs are mediated by a specific immunological mechanism, being induced by a single group of NSAIDs with patients tolerating other chemically unrelated NSAIDs. This category includes two different phenotypes: single-NSAID-induced urticaria/angioedema or anaphylaxis (SNIUAA), mediated by specific IgE antibodies, and single-NSAID-induced delayed reactions (SNIDR), where a T-cell-mediated mechanism is involved (Kowalski et al., 2013).

CRs constitute the most frequent group of HDRs in both adults and children. Among these, the NSAID-induced urticaria/angioedema (NIUA) phenotype accounts for more than 60% of CR cases (Doña et al., 2011; Zambonino et al., 2013). The development of *in vitro* diagnostic tests is hampered by the poor understanding of the underlying mechanism of CRs. Thus, drug provocation remains to date the gold standard procedure to confirm the diagnosis, but it implies risks and requires specific facilities (Pérez-Sánchez et al., 2020). This highlights the necessity of unveiling the mechanisms underlying CRs to NSAIDs, and to characterize biomarkers that help improve the diagnosis of HDRs. Pharmacogenetic studies have investigated the genetic factors contributing to HDRs associated with NSAIDs use. Several studies have examined the relationship between variants in genes involved in the mechanism of action of NSAIDs and the resulting imbalance between leukotrienes and prostaglandins (Ayuso et al., 2015; Plaza-Serón et al., 2018; Cornejo-García et al., 2012; Cornejo-García et al., 2016; Jurado-Escobar et al., 2021a; Plaza-Serón et al., 2016; García-Martín et al., 2021; Jurado-Escobar et al., 2021b). Their findings have shown several variants weakly associated with the susceptibility toNSAIDs HRs. Moreover, supporting the hypothesis that additional mechanisms may contribute to the underlying pathophysiology of these reactions, a range of candidate gene studies and genome-wide association studies have investigated the role of genetic variants in pathways related to inflammation, mast cell activation signaling, NSAIDs metabolism, and the major histocompatibility complex (Plaza-Serón et al., 2018; Mayorga et al., 2019; Amo et al., 2016a).

However, the available evidence remains insufficient to achieve a comprehensive understanding of the mechanisms involved in the development of NSAIDs-HDRs and recent investigations suggest additional contributors (Ayu et al., 2021). Increasing evidence highlights the influence of vitamin D and the immune system. Vitamin D deficiency influences the pathogenesis of asthma (Pfeffer et al., 2018), respiratory diseases (Palikhe NS. et al., 2008), and severe cutaneous HDRs (Pholmoo et al., 2024). Therefore, genes involved in those mechanisms represent a main target of study. Thus, SNVs in *FCER1A, FCER1B, FCER1G,* and *IL4* genes have been associated with asthma (Palikhe NS. et al., 2008; Ya et al., 2014) and allergic diseases (Liao et al., 2015; Liu et al., 2011). Moreover, new insights into the role of vitamin D in the immune system have elucidated its importance as an immunomodulator. Hence, variants in genes related to its metabolism, mechanism of action, and signaling pathways have been associated with asthma, mainly those located in interleukin genes regulated by vitamin D, such as IL4 and its receptor (Li et al., 2011; Hutchinson et al., 2018; Pillai et al., 2011; Leung et al., 2015; Zhang et al., 2015). Furthermore, in a previous NGS study in the promoter area of sixteen genes of the vitamin D pathway and the FCɛRI, we identified a strong association between the promoter *FCER1G* rs36233990 single nucleotide variant (SNV) and IgE-mediated HRs to NSAIDs and ß-lactam antibiotics (Amo et al., 2019). Also, SNVs in the vitamin receptor gene have been associated with the response to antiviral therapy in patients with chronic hepatitis and other disorders (García-Martín et al., 2013; Jiménez-Jiménez et al., 2021). From a mechanistic point of view, vitamin D receptors have been identified in different immune cells and their actions inhibit the production of T helper lymphocytes (Baeke et al., 2010) and decrease the production of pro-inflammatory cytokines (Hansdottir et al., 2010; Holick, 2011). The vitamin D pathway comprises several Cytochrome P450 (CYP450) enzymes, transporters such as the vitamin binding protein (GC), and receptors such as vitamin D receptor (VDR) and retinoid receptor X (RXR), and interleukins such as IL4 and IL13 (Amo et al., 2019). Regarding NSAIDs HRs which are mediated by a specific immune mechanism, the interaction between IgE and the FCɛRI triggers the activation of basophils and mast cells, leading to the release of inflammatory mediators that coordinate the immune response (Kinet, 2003). Despite different studies having identified genetic associations with HDRs (Palikhe NS. et al., 2008; Palikhe N. et al., 2008; Bae et al., 2007; Choi et al., 2005), extensive analysis of polymorphisms associated with these pathologies is lacking.

This study aims to investigate the genetic basis of hypersensitivity reactions (HRs) to non-steroidal anti-inflammatory drugs (NSAIDs) by analyzing SNVs located in the exonic and 3’ untranslated regions of genes involved in the vitamin D pathway and the high-affinity IgE receptor. Specifically, the study focuses on identifying genetic variants associated with two major categories of NSAID hypersensitivity: CR and SNIUAA. Additionally, we evaluated the functional impact of these variants by examining their correlation with serum levels of vitamin D and IgE. By integrating genetic and biochemical data, the objective is to uncover potential biomarkers that could enhance the understanding, diagnosis, and clinical management of NSAID-induced hypersensitivity reactions.

## 2 Materials and methods

### 2.1 Study population

We included a total cohort of 1,962 individuals, composed of 314 SNIUAA patients, 668 patients with CR NSAIDs-HDRs, and 980 tolerant individuals to NSAIDs. All participants were of Caucasian Spanish origin. Tolerant individuals were 516 patients with allergic rhinitis with or without asthma, and 464 healthy controls without an history of atopy or ADRs; these two groups were also designated as atopic and non-atopic controls, respectively. All participants gave written consent for participation. Patients were recruited at Hospitals participating in the study, whereas tolerant individuals were selected among students and staff in the University and Hospitals participating in the study. Table 1 summarizes the characteristics of the different study groups. The inclusion criteria for participants and details for diagnosis are described elsewhere (Pérez-Sánchez et al., 2020; Amo et al., 2016b; Kowalski et al., 2019). The protocol for this study was in accordance with the Declaration of Helsinki and its subsequent revisions and was approved by the respective Ethics Committees of the participating Hospitals.

**TABLE 1:** Characteristics of the study population.

	SNIUAA (N = 314)	CR NSAIDs (N = 668)
Women (%)	206 (65.6)	391 (58.7)
Age [Mean; range]	45.4 [5-82]	41.8 [5-92]
History of atopy (N,%)	66 (21.0)	149 (22.4)
History of urticaria (N,%)	4 (1.3)	4 (0.6)
Cutaneous symptoms (N, %)	170 (54.1)	441 (66.2)
Respiratory symptoms (N, %)	5 (1.6)	79 (11.9)
Anaphylaxis (N, %)	108 (34.4)	49 (7.4)
Blended reactions (N, %)	9 (2.9)	82 (12.3)
Others (N, %)	20 (6.4)	11 (1.7)
Unknown (N, %)	2 (0.6)	4 (0.6)

Blended reactions: cutaneous and respiratory symptoms.

Others: exanthema, erythema, eczema, glottic edema, rash or pruritus.

### 2.2 Identification of genetic variants using NGS

To identify novel gene variants, a randomly selected subset of 131 participants was analyzed. This cohort included 46 CR patients with NSAIDs HRs, 41 SNIUAA patients, and 44 individuals tolerant to NSAIDs (22 atopic controls and 22 non-atopic controls). Characteristics of these study groups are summarized in Supplementary Table S1. Specific enrichment was developed using Haloplex design to prepare sequencing libraries for NGS. The sequenced areas included all exons and the flanking exonic regions of the genes *FCER1A, MS4A2, FCER1G, VDR, GC, CYP2R1, CYP27A1, CYP27B1, CYP24A1, RXRA, RXRB, RXRG, IL4, IL4R, IL13* and *IL13RA1*, as well as the 3’UTR region of these genes. Details of the sequenced areas are included in Supplementary Table S2. Genomic DNA extraction and the sequencing analysis were conducted as described elsewhere (Amo et al., 2019).

### 2.3 Replication analyses

This phase included all the patients and individuals recruited in the study. After sequencing analysis, single-analyzed SNVs were selected according to different inclusion and exclusion criteria (further details are in the results section). The SNVs were genotyped in triplicate by using SNV TaqMan assays (Life Technologies S.A., Alcobendas, Madrid, Spain) as described elsewhere (Amo et al., 2019). The procedure was carried out using the manufacturer’s instructions.

### 2.4 Determination of IgE

The determination of total serum IgE in hypersensitivity patients to NSAIDs and individuals tolerant to NSAIDs was conducted using the ImmunoCAP system-FEIA (Pharmacia, Uppsala, Sweden) All samples were collected during individual evaluations at the allergy departments of the participating hospitals and were analyzed in triplicate according to the instructions provided by the manufacturer (Campo et al., 2013).

### 2.5 Measurement of vitamin D

. Vitamin D was quantitatively determined by using the immunoassay 25-Hydroxy Vitamin D^S^ EIA (Immunodiagnostic Systems LTD, United Kingdom) according to the instructions provided by the manufacturer. All samples were collected during individual evaluations at the allergy departments of the participating hospitals and were analyzed in triplicate according to the instructions provided by the manufacturer (Jiménez-Jiménez et al., 2021) (see Supplementary Material).

### 2.6 Statistical analysis

Quantitative variables were summarized as means with standard deviations, and categorical variables were expressed as frequencies. Group comparisons were performed by the chi-squared test for sex; and Kruskal–Wallis test (IBM SPSS Statistics for Windows, Version 22.0) for age, Ige levels and vitamin D levels. Box plots illustrated the distribution of IgE and vitamin D levels across study groups, with pairwise comparisons performed using Dunn’s test and Bonferroni correction to control the error rate.

To calculate allele and genotypic frequencies, the Hardy-Weinberg equation and to determine differences between groups, the R package SNPassoc was used (GonzáLez et al., 2007). The association between SNVs and groups was estimated by logistic regression adjusted by sex, including odds ratio (OR) with a 95% confidence interval (CI) or by Relative Risk (RR) when the variation was not found in the reference group. The association between SNVs and quantitative traits (IgE levels and vitamin D levels) was assessed by generalized linear regression models adjusted by sex. Also, multiple comparison adjustments were carried out using the Bonferroni correction (Bland and Altman, 1995) and the Benjamini–Hochberg (BH) procedure to control the False Discovery Rate (FDR). The differences were considered statistically significant when *P*-values under 0.05 were observed.

## 3 Results

### 3.1 Identification of gene variants using NGS and TaqMan genotyping

We applied targeted NGS to investigate novel variants in a subset of 131 participants. We identified 216 variants situated in the exons and 3′region of the aforementioned genes, including 186 variants that were not previously reported. Details are summarized in Supplementary Table S3. Afterward, the replication phase was carried out in two stages. Firstly, a selection of the SNVs identified using NGS was conducted according to the following criteria, i) Those SNVs with a minor allele frequency (MAF) > 5%, or previously reported in pharmacogenetic studies or that showed differences after group comparison, or an association with vitamin D or IgE serum levels (p-value <0,1), ii) Those SNVs with a MAF >1% with a functional effect. iii) Those nonsynonymous SNVs with a MAF <5% and previously reported in pharmacogenetic studies. SNVs were excluded from replication if they exhibited strong linkage disequilibrium, contained more than one nucleotide change, or could not be genotyped because of failure to design a TaqMan assay. Thus, 42 SNVs in the genes *MS4A2, FCER1G, VDR, GC, CYP2R1, CYP27A1, CYP27B1, CYP24A1, RXRB, RXRG, IL4, IL4R,* and *IL13* were genotyped in 200 CR patients with NSAIDs HRs, 200 SR patients and 200 non-atopic tolerant individuals. The genetic association analysis is shown in Table 2. Allele frequencies for Vitamin D-related genes and IgE-related genes are consistent with those reported by our group in Spanish individuals (García-Martín et al., 2023; Agún et al., 2022; Mateos-Munõz et al., 2016; Jimenez-Jimenez et al., 2015; Gar et al., 2013).

**TABLE 2:** P-values obtained from the combined data analyses of the first stage of the replication phase (N ≈ 600).

	SNIUAA-non atopic controls			CR NSAIDs-Non atopic controls			SNIUAA-CR NSAIDs			Vit D level	IgE level
	Cod	Dom	Rec	Cod	Dom	Rec	Cod	Dom	Rec	Cod (KW)	Cod (KW)
*FCER1G*											
rs11421	0.64	0.36	0.99	0.68	0.40	0.64	0.91	0.89	0.72	0.82	0.89
rs7528588	0.88	0.67	0.70	1.00	0.94	0.95	0.83	0.62	0.64	0.45	0.53
rs4503368	0.58	0.89	0.36	0.49	0.47	0.50	0.26	0.62	0.19	0.36	0.55
*RXRG*											
rs2134095	0.26	0.60	**0.10**	0.80	0.86	0.51	0.57	0.83	0.29	0.52	0.94
rs1128977	**0.01**	**0.02**	0.41	0.41	0.26	0.82	0.34	0.24	0.63	0.11	0.14
rs113471	0.34	0.22	0.26	0.89	0.62	0.89	0.55	0.46	0.34	0.41	0.37
*CYP27A1*											
rs151203082	0.16	n.a	n.a	0.92	n.a	n.a	0.15	n.a	n.a	0.95	0.90
*GC*											
rs139523630	0.09	n.a	n.a	**0.05**	**0.02**	0.25	0.40	0.44	0.21	0.89	**0.003**
rs4588	0.84	0.58	0.98	0.75	0.81	0.44	0.63	0.72	0.46	0.45	0.70
rs7041	**0.07**	0.24	**0.02**	0.35	0.16	0.48	0.67	0.65	0.39	0.53	0.79
*IL13*											
rs20541	0.36	0.26	0.23	0.58	0.67	0.30	0.77	0.49	0.96	0.93	0.88
rs1295685	0.30	0.15	0.96	0.82	0.64	0.83	0.60	0.33	0.97	0.77	0.79
rs848	0.40	0.19	0.44	0.80	1.00	0.54	0.38	0.19	0.97	0.44	0.81
rs847	0.35	0.15	0.48	0.79	0.56	0.59	0.66	0.39	0.97	0.74	0.52
rs143032763	0.54	n.a	n.a	0.25	n.a	n.a	0.60	n.a	n.a	0.61	0.02
*IL4*											
rs35648164	**0.09**	n.a	n.a	**0.01**	n.a	n.a	0.42	n.a	n.a	0.51	0.42
rs2070874	0.28	0.14	0.32	0.88	0.70	0.66	0.57	0.29	0.66	0.16	0.20
rs2243291	**0.03**	0.17	**0.01**	0.27	0.76	0.11	0.54	0.35	0.40	**0.07**	0.45
*RXRB*											
rs2744537	0.41	0.36	0.52	0.13	0.90	**0.05**	0.36	0.34	0.20	0.75	**0.02**
rs6531	0.26	0.24	0.47	0.15	0.78	**0.08**	0.48	0.37	0.31	0.94	**0.03**
*CYP2R1*											
rs117913124	0.90	n.a	n.a	0.57	n.a	n.a	0.76	n.a	n.a	0.21	0.40
rs12794714	**0.08**	**0.04**	**0.10**	0.28	0.15	0.25	0.96	0.82	0.79	0.56	0.22
*MS4A2*											
rs569108	0.66	n.a	n.a	**0.08**	n.a	n.a	0.19	n.a	n.a	0.59	0.43
rs512555	0.71	n.a	n.a	**0.06**	n.a	n.a	0.14	n.a	n.a	0.82	0.65
VDR											
rs78783628	0.08	0.13	0.03	0.86	0.59	0.91	**0.08**	**0.09**	**0.05**	0.17	0.90
rs3847987	**0.07**	0.79	**0.02**	0.33	0.73	0.21	0.59	0.64	0.32	**0.10**	0.29
rs739837	**0.02**	**0.08**	**0.01**	0.58	0.91	0.34	**0.06**	0.22	**0.02**	0.11	0.87
rs731236	0.18	0.28	**0.08**	0.45	0.33	0.63	**0.08**	**0.03**	0.19	0.26	0.62
rs2228570	0.86	0.59	0.78	0.35	0.16	0.45	0.59	0.35	0.51	**0.05**	0.98
CYP27B1											
rs8176344	0.76	n.a	n.a	0.81	n.a	n.a	0.84	n.a	n.a	0.79	0.20
IL4R											
rs17548704	0.74	n.a	n.a	0.75	n.a	n.a	0.99	n.a	n.a	0.78	0.57
rs1805010	0.79	0.94	0.51	0.31	0.26	0.17	0.11	0.22	**0.04**	**0.08**	**0.04**
rs6413500	0.59	n.a	n.a	0.74	n.a	n.a	0.86	n.a	n.a	0.49	0.54
rs1805011	0.25	0.43	0.10	0.98	0.93	0.85	0.35	0.95	0.15	0.18	0.46
rs1805013	0.33	0.14	0.82	0.26	0.29	0.27	**0.003**	**0.003**	0.27	0.13	0.92
rs1805015	0.32	0.39	0.16	0.67	0.39	0.99	0.18	0.11	0.20	0.36	0.87
rs1801275	0.14	**0.09**	0.15	0.58	0.40	0.40	0.93	0.72	0.97	0.31	0.20
rs3024678	0.42	n.a	n.a	**0.09**	n.a	n.a	0.28	n.a	n.a	0.11	0.76
rs1049631	0.91	0.88	0.66	0.14	0.38	**0.05**	0.27	0.54	**0.10**	0.74	0.74
rs8832	0.43	0.19	0.60	0.51	0.99	0.27	0.29	0.32	0.14	0.23	0.77
rs1029489	0.53	0.27	0.87	0.53	0.97	0.28	0.50	0.45	0.26	0.47	0.53
CYP24A1											
rs2762934	**0.09**	0.21	**0.03**	0.66	0.46	0.45	0.58	0.78	0.30	0.99	0.93

Cod: codominant; Dom: dominant; Rec: recessive.

P-values in bold (p value <0,1) were included in the second stage of the replication phase.

Those SNVs that showed statistically significant associations with a p-value <0,1 were included in the second stage of the replication phase. Thus, twenty-two SNVs in *RXRG, GC, IL13, IL4, RXRB, CYP2R1, MS4A2, VDR, IL4R,* and *CYP24A1* genes were genotyped in a total cohort of 1962 individuals. In addition, the SNVs *FCER1G* rs11421*, GC* rs4588*, CYP27B1* rs8176344, and *IL4R* rs1029489 were also included in the second stage. These SNVs were previously studied by our group (Amo et al., 2016a; García-Martín et al., 2023). Thus, the SNV *FCER1G* rs11421 has shown an association with the total serum IgE levels in CR NSAIDs hypersensitivity patients with respiratory symptoms (Palikhe NS. et al., 2008) whereas *IL4R* rs1029489 has been associated with decreased exacerbation rates in asthmatic patients undergoing treatment with an anti-IL-4 receptor α antagonist (Slager et al., 2012). Additionally, the SNV *GC* rs4588 has been related to vitamin D levels (Jiang et al., 2018) and the *CYP27B1* rs8176344 mutant variant is associated with increased CYP27B1 activity (Jacobs et al., 2013). Table 3 summarizes the SNVs included in the second stage, including their location, the associated effect, MAF, and the p-values associated with the Hardy-Weinberg equilibrium test of these variants in each studied group. The observed frequencies were in concordance with the results previously described in the 1,000 Genomes public database (Browse a Genome, 2024).

**TABLE 3:** SNVs analysed in the second stage of the replication phase.

					Non atopic controls		SNIUAA		CR NSAIDs		Atopic controls		MAF (1000 Genomes)	
Gene	SNV	Minor allele	Chromosomal location	Area/Effect	MAF	HWE p-value	MAF	HWE p-value	MAF	HWE p-value	MAF	HWE p-value	Global	European
*FCER1G*	rs11421	C	1:161188936	Region 3′	0.178	0.34	0.168	0.68	0.178	0.50	0.174	0.88	0.196C	0.147C
*RXRG*	rs1128977	A	1: 165389129	Ala17	0.342	0.11	0.355	0.32	0.365	0.37	0.344	0.10	0.227A	0.400A
*GC*	rs139523630	C	4:72606530	Region 3′	0.008	1.00	0.015	1.00	0.014	0.08	0.011	1.00	0.003C	0.006C
*GC*	rs4588	T	4:72618323	Thr436Lys	0.326	0.16	0.306	0.59	0.295	0.50	n.a	n.a	0.208T	0.248T
*GC*	rs7041	A	4:72618334	Asp432Glu	0.516	0.37	0.452	0.65	0.455	0.63	n.a	n.a	0.382C	0.417A
*IL13*	rs143032763	G	5: 131996752	Región 3′	0.011	1.00	0.012	1.00	0.014	1.00	0.005	1.00	0.002G	0.005G
*IL4*	rs35648164	T	5: 132015555	Asp111	0.014	1.00	0.008	1.00	0.004	1.00	0.007	1.00	0.005T	0.012T
*IL4*	rs2243291	C	5: 132018983	Region 3′	0.137	1.00	0.182	0.44	0.163	1.00	0.164	0.13	0.447C	0.177C
*RXRB*	rs2744537	A	6: 33162215	Region 3′	0.239	0.06	0.250	0.65	0.246	0.32	0.234	0.89	0.131A	0.281A
*RXRB*	rs6531	G	6: 33163451	Phe384	0.234	**0.01**	0.246	0.45	0.249	0.54	0.233	0.89	0.142G	0.281G
*CYP2R1*	rs12794714	A	11:14913575	Ser59	0.426	0.56	0.490	0.74	0.453	0.19	n.a	n.a	0.349A	0.447A
*MS4A2*	rs569108	G	11:59863104	Glu237Gly	0.038	1.00	0.039	0.37	0.028	0.39	0.037	1.00	0.112G	0.037G
*MS4A2*	rs512555	T	11:59863253	Region 3′	0.037	1.00	0.038	0.37	0.030	0.46	0.036	1.00	0.089T	0.037T
*VDR*	rs78783628	-	12:48237736	Region 3′	0.551	1.00	0.471	0.57	0.536	0.53	0.546	0.47	0.499A	0.478A
*VDR*	rs3847987	A	12: 48238068	Region 3′	0.120	**0.004**	0.107	0.76	0.117	0.50	0.136	0.40	0.129A	0.146A
*VDR*	rs739837	G	12:48238221	Region 3′	0.436	0.72	0.530	0.57	0.470	0.80	0.463	0.92	0.494T	0.446G
*VDR*	rs731236	G	12:48238757	Ile352	0.419	0.73	0.350	0.06	0.387	0.67	0.402	0.36	0.277G	0.400G
*VDR*	rs2228570	A	12:48272895	Thr1Met	0.364	0.91	0.368	0.54	0.388	0.39	0.333	1.00	0.328A	0.378A
*CYP27B1*	rs8176344	G	12:58159173	Val166Leu	0.003	1.00	0.008	1.00	0.010	**0.02**	n.a	n.a	0.015G	0.001G
*IL4R*	rs1805010	G	16:27356203	Ile75Val	0.438	0.90	0.414	0.62	0.442	0.90	n.a	n.a	0.456G	0.425G
*IL4R*	rs1805013	T	16: 27373980	Ser436Leu	0.044	0.14	0.021	0.12	0.039	1.00	0.043	1.00	0.039	0.058
*IL4R*	rs1801275	G	16:27374400	Gln551Arg	0.238	0.64	0.162	1.00	0.198	0.59	0.219	0.51	0.375G	0.208G
*IL4R*	rs3024678	T	16: 27374696	Pro675Ser	0.026	**0.02**	0.019	1.00	0.011	1.00	0.018	0.13	0.005T	0.015T
*IL4R*	rs1049631	G	16: 27375542	Region 3′	0.447	1.00	0.421	0.91	0.457	0.41	0.435	0.62	0.430A	0.443G
*IL4R*	rs1029489	A	16:27376217	Region 3′	0.415	0.62	0.368	0.68	0.393	0.31	n.a	n.a	0.456G	0.398A
*CYP24A1*	rs2762934	A	20:52771261	Region 3′	0.268	0.19	0.212	0.13	0.250	0.65	0.220	0.08	0.189A	0.223A

### 3.2 Determination of genetic variants associated with NSAID hypersensitivity reactions

Table 1 summarizes the characteristics of the different NSAID HRs study groups. In addition, the analysis included 464 non-atopic tolerant individuals (62.7% female; mean age: 22.1 years, range: 19–58) and 516 atopic tolerant individuals (55.2% female; mean age: 32.2 years, range: 14–79). Overall comparisons revealed statistically significant differences in sex (*P*-value: 0.045) and age (*P*-value <0.001) across all groups included in the study.

NSAIDs HRs can be distinguished into two main groups according to the underlying mechanism, CR and SR. Therefore, we investigated the association between SNVs in genes related to vitamin D and FCɛRI in the two main categories of NSAIDs HRs, specifically in SNIUAA and CR NSAIDs HRs.

### 3.3 Genetic association study in single-NSAID-induced urticaria/angioedema or anaphylaxis patients (SNIUAA)

The results of the binary logistic regression are shown in Table 4. After adjustment for multiple comparisons according to each genetic model, four variants remained statistically associated: the SNVs *VDR* rs78783628 and rs739837, located at 3′region, the synonymous SNV *VDR* rs731236, and the SNV placed at *CYP24A1* 3′region rs2762934, (Table 4).

**TABLE 4:** SNVs significantly associated with SNIUAA patients (adjusted by sex). (C: Codominant model, D: Dominant model, and R: Recessive model).

		SNIUAA vs. non-atopic controls				SNIUAA vs. atopic controls			SNIUAA vs. controls		
Gene/SNV	Genotype	SNIUAA (N, Freq.)	Non atopic controls (N, Freq.)	OR (95% CI)	p-value (crude/BH)	Atopic controls (*) (N, Freq.)	OR (95% CI)	p-value (crude/BH)	Controls (N, Freq.)	OR (95% CI)	p-valor (crude/BHBH)
*GC*											
rs7041											
CD	C/C	92 (0.293)	69 (0.22)	1.00		n.a	n.a	n.a	n.a	n.a	n.a
	C/A	160 (0.51)	166 (0.53)	0.72 (0.49–1.06)	0.096/0.512	n.a	n.a	n.a	n.a	n.a	n.a
	A/A	62 (0.198)	78 (0.249)	0.59 (0.38–0.94)	0.026/0.302	n.a	n.a	n.a	n.a	n.a	n.a
D	C/C	92 (0.293)	69 (0.22)	1.00		n.a	n.a	n.a	n.a	n.a	n.a
	C/A-A/A	222 (0.707)	244 (0.78)	0.68 (0.48–0.98)	0.038/0.238	n.a	n.a	n.a	n.a	n.a	n.a
R	C/C-C/A	252 (0.802)	235 (0.751)	1.00		n.a	n.a	n.a	n.a	n.a	n.a
	A/A	62 (0.198)	78 (0.249)	0.74 (0.51–1.08)	0.117/0.438	n.a	n.a	n.a	n.a	n.a	n.a
*CYP2R1*											
rs12794714						n.a	n.a	n.a	n.a	n.a	n.a
CD	G/G	83 (0.264)	105 (0.339)	1.00		n.a	n.a	n.a	n.a	n.a	n.a
	G/A	154 (0.49)	146 (0.471)	1.33 (0.92–1.92)	0.127/0.569	n.a	n.a	n.a	n.a	n.a	n.a
	A/A	77 (0.245)	59 (0.19)	1.65 (1.06–2.58)	0.027/0.302	n.a	n.a	n.a	n.a	n.a	n.a
D	G/G	83 (0.264)	105 (0.339)	1.00		n.a	n.a	n.a	n.a	n.a	n.a
	G/A-A/A	231 (0.736)	205 (0.661)	1.42 (1.01–2.01)	0.044/0.238	n.a	n.a	n.a	n.a	n.a	n.a
R	G/G-G/A	237 (0.755)	251 (0.81)	1.00		n.a	n.a	n.a	n.a	n.a	n.a
	A/A	77 (0.245)	59 (0.19)	1.38 (0.94–2.03)	0.095/0.438	n.a	n.a	n.a	n.a	n.a	n.a
*VDR*											
rs78783628											
CD	−/−	71 (0.231)	94 (0.302)	1.00		146 (0.3)	1.00		240 (0.301)	1.00	
	A/-	148 (0.48)	154 (0.495)	1.27 (0.87–1.86)	0.047/0.411	235 (0.482)	1.28 (0.90–1.83)	0.101/0.462	389 (0.488)	1.28 (0.93–1.78)	0.041/0.286
	A/A	89 (0.289)	63 (0.203)	1.89 (1.21–2.96)	0.005/0.134	106 (0.218)	1.72 (1.15–2.57)	0.008/0.157	169 (0.212)	1.79 (1.24–2.59)	0.002/0.058
D	−/−	71 (0.231)	94 (0.302)	1.00		146 (0.3)	1.00		240 (0.301)	1.00	
	A/--A/A	237 (0.77)	217 (0.698)	1.45 (1.01–2.08)	0.042/0.238	341 (0.7)	1.42 (1.02–1.97)	0.036/0.273	558 (0.699)	1.44 (1.06–1.95)	0.019/0.160
R	−/−A/-	219 (0.711)	248 (0.797)	1.00		381 (0.782)	1.00		629 (0.788)	1.00	
	A/A	89 (0.289)	63 (0.203)	1.62 (1.12–2.35)	0.011/0.119	106 (0.218)	1.46 (1.05–2.03)	0.023/0.121	169 (0.212)	1.53 (1.13–2.06)	0.006/0.050
rs739837											
CD	T/T	70 (0.229)	89 (0.31)	1.00		125 (0.283)	1.00		214 (0.294)	1.00	
	T/G	147 (0.482)	145 (0.505)	1.30 (0.88–1.91)	0.192/0.614	220 (0.499)	1.18 (0.82–1.70)	0.360/0.849	365 (0.501)	1.23 (0.89–1.72)	0.214/0.621
	G/G	88 (0.289)	53 (0.185)	2.15 (1.35–3.43)	0.001/0.075	96 (0.218)	1.65 (1.09–2.50)	0.017/0.226	149 (0.205)	1.84 (1.26–2.69)	0.002/0.058
D	T/T	70 (0.229)	89 (0.31)	1.00		125 (0.283)	1.00		214 (0.294)	1.00	
	T/G-G/G	235 (0.77)	198 (0.69)	1.52 (1.06–2.20)	0.024/0.238	316 (0.717)	1.33 (0.94–1.86)	0.103/0.454	514 (0.706)	1.41 (1.03–1.92)	0.029/0.203
R	T/T-T/G	217 (0.712)	234 (0.815)	1.00		345 (0.782)	1.00		579 (0.795)	1.00	
	G/G	88 (0.289)	53 (0.185)	1.82 (1.23–2.68)	0.002/0.054	96 (0.218)	1.48 (1.06–2.07)	0.022/0.121	149 (0.205)	1.60 (1.18–2.18)	0.003/0.032
rs731236											
CD	A/A	123 (0.398)	102 (0.332)	1.00		183 (0.368)	1.00		285 (0.355)	1.00	
	A/G	156 (0.505)	154 (0.502)	0.82 (0.58–1.16)	0.258/0.642	230 (0.463)	0.99 (0.73–1.34)	0.943/1.000	384 (0.478)	0.92 (0.69–1.22)	0.573/0.898
	G/G	30 (0.097)	51 (0.166)	0.48 (0.28–0.81)	0.006/0.134	84 (0.169)	0.53 (0.33–0.85)	0.009/0.157	135 (0.168)	0.51 (0.32–0.80)	0.003/0.065
D	A/A	123 (0.398)	102 (0.332)	1.00		183 (0.368)	1.00		285 (0.355)	1.00	
	A/G-G/G	186 (0.602)	205 (0.668)	0.73 (0.53–1.02)	0.067/0.258	314 (0.632)	0.87 (0.65–1.16)	0.336/0.756	519 (0.645)	0.81 (0.62–1.07)	0.139/0.368
R	A/A-A/G	279 (0.903)	256 (0.834)	1.00		413 (0.831)	1.00		669 (0.832)	1.00	
	G/G	30 (0.097)	51 (0.166)	0.54 (0.33–0.87)	0.011/0.119	84 (0.169)	0.53 (0.34–0.83)	0.004/0.084	135 (0.168)	0.53 (0.35–0.81)	0.002/0.032
*IL4R*											
rs1805013											
CD	C/C	298 (0.961)	329 (0.916)	1.00		380 (0.916)	1.00		709 (0.916)	1.00	
	C/T	11 (0.035)	28 (0.078)	0.43 (0.21–0.88)	0.020/0.280	35 (0.084)	0.39 (0.20–0.79)	0.009/0.157	63 (0.081)	0.41 (0.21–0.78)	0.007/0.122
	T/T	1 (0.003)	2 (0.006)	0.52 (0.05–5.82)	0.599/0.881	0 (0)	NA (0.00-NA)	1.000/1.000	2 (0.003)	1.06 (0.10–11.81)	0.959/1.000
D	C/C	298 (0.961)	329 (0.916)	1.00		380 (0.916)	1.00		709 (0.916)	1.00	
	C/T-T/T	12 (0.039)	30 (0.084)	0.43 (0.22–0.87)	0.013/0.238	35 (0.084)	0.43 (0.22–0.84)	0.009/0.189	65 (0.084)	0.43 (0.23–0.80)	0.004/0.056
R	C/C-C/T	309 (0.997)	357 (0.994)	1.00		415 (1)	1.00		772 (0.997)	1.00	
	T/T	1 (0.003)	2 (0.006)	0.55 (0.05–6.13)	0.629/0.719	0 (0)	NA (0.00-NA)	0.220/0.544	2 (0.003)	1.13 (0.10–12.49)	0.923/0.937
rs1801275											
CD	A/A	213 (0.703)	182 (0.587)	1.00		293 (0.61)	1.00		475 (0.601)	1.00	
	A/G	82 (0.271)	109 (0.352)	0.64 (0.45–0.90)	0.011/0.205	161 (0.335)	0.71 (0.52–0.98)	0.038/0.301	270 (0.342)	0.68 (0.51–0.92)	0.011/0.137
	G/G	8 (0.026)	19 (0.061)	0.35 (0.15–0.82)	0.015/0.240	26 (0.054)	0.45 (0.20–1.03)	0.057/0.381	45 (0.057)	0.40 (0.19–0.87)	0.021/0.203
D	A/A	213 (0.703)	182 (0.587)	1.00		293 (0.61)	1.00		475 (0.601)	1.00	
	A/G-G/G	90 (0.297)	128 (0.413)	0.59 (0.42–0.83)	0.002/0.108	187 (0.39)	0.68 (0.50–0.92)	0.014/0.196	315 (0.399)	0.64 (0.48–0.85)	0.002/0.056
R	A/A-A/G	295 (0.974)	291 (0.939)	1.00		454 (0.946)	1.00		745 (0.943)	1.00	
	G/G	8 (0.026)	19 (0.061)	0.40 (0.17–0.94)	0.028/0.252	26 (0.054)	0.51 (0.23–1.14)	0.083/0.349	45 (0.057)	0.46 (0.21–0.98)	0.029/0.158
*CYP24A1*											
rs2762934											
CD	G/G	188 (0.605)	170 (0.55)	1.00		295 (0.632)	1.00		465 (0.599)	1.00	
	G/A	114 (0.367)	112 (0.362)	0.92 (0.66–1.29)	0.643/0.907	142 (0.304)	1.24 (0.91–1.69)	0.173/0.626	254 (0.327)	1.10 (0.84–1.46)	0.488/0.897
	A/A	9 (0.029)	27 (0.087)	0.30 (0.14–0.65)	0.002/0.075	30 (0.064)	0.46 (0.21–0.99)	0.047/0.341	57 (0.073)	0.38 (0.19–0.79)	0.010/0.137
D	G/G	188 (0.605)	170 (0.55)	1.00		295 (0.632)	1.00		465 (0.599)	1.00	
	G/A-A/A	123 (0.395)	139 (0.45)	0.80 (0.58–1.10)	0.175/0.465	172 (0.368)	1.10 (0.82–1.48)	0.519/0.775	311 (0.401)	0.97 (0.74–1.27)	0.831/0.975
R	G/G-G/A	302 (0.971)	282 (0.913)	1.00		437 (0.936)	1.00		719 (0.927)	1.00	
	A/A	9 (0.029)	27 (0.087)	0.31 (0.14–0.66)	0.001/0.054	30 (0.064)	0.43 (0.20–0.91)	0.019/0.121	57 (0.073)	0.37 (0.18–0.76)	0.003/0.032
	C/C	54 (0.184)	56 (0.223)	0.79 (0.52–1.20)	0.261/0.522	82 (0.185)	0.99 (0.68–1.45)	0.955/0.972	138 (0.199)	0.91 (0.64–1.29)	0.583/0.765

BH:Benjamini–Hochberg correction.

*Some SNVs, could not be determined in the group of controls due to DNA, shortage these are labelled in the table as not available (n.a.).

SNIUAA patients were categorized according to their symptoms. Our findings demonstrated that SNIUAA patients carrying the *CYP2R1* rs12794714 (*P*-value: 0.039; OR: 0.58) mutant allele, or the *CYP24A1* rs2762934 mutant allele in homozygosis (*P*-value: 0.041; OR: 0.22) manifested less risk of developing isolated cutaneous symptoms which include urticaria and/or angioedema (Supplementary Table S4). Moreover, SNIUAA patients carrying the *GC* rs139523630 mutant allele or the *CYP24A1* rs2762934 mutant allele manifested a higher risk of developing anaphylaxis, whereas those carrying the *IL4* rs2243291 mutant allele in homozygosis or the *IL4R* rs1805013 mutant allele showed less risk of suffering anaphylaxis (Supplementary Table S5). However, after correcting for multiple comparisons under each genetic model, none of these SNVs retained statistical differences.

### 3.4 Identification of genetic variants associated with cross-reactive NSAIDs hypersensitivity reactions

To study the association between SNVs and CR NSAIDs HRs, the genotypic frequencies of twenty-six SNVs were analyzed in 649 patients with CR, 369 non-atopic tolerant individuals, and 501 atopic tolerant individuals to NSAIDs. The analysis revealed statistically significant differences between CR NSAIDs HRs patients and non-atopic tolerant individuals. Moreover, statistically significant differences between CR NSAIDs HRs patients and atopic-tolerant individuals were observed (Table 5). However, none of these SNVs remained statistically associated after adjustment for multiple comparisons according to each genetic model.

**TABLE 5:** SNVs significantly associated with CR NSAIDs patients (adjusted by sex). (C: Codominant model, D: Dominant model, and R: Recessive model).

		CR NSAIDs vs. non-atopic controls				CR NSAIDs vs. atopic controls*			CR NSAIDs vs. controls		
Gene/SNV	Genotype	CR NSAIDs (N, Freq.)	Controls NA (N, Freq.)	OR (95% CI)	p-value (crude/BH)	Atopic controls (N, Freq.)	OR (95% CI)	p-value (crude/BH)	Controls (N, Freq.)	OR (95% CI)	p-value (crude/BH)
*GC*											
rs4588											
CD	G/G	307 (0.501)	138 (0.437)	1.00		n.a	n.a	n.a	n.a	n.a	n.a
	G/T	249 (0.406)	150 (0.475)	0.74 (0.56–0.99)	**0.042/0.693**	n.a	n.a	n.a	n.a	n.a	n.a
	T/T	57 (0.093)	28 (0.089)	0.91 (0.56–1.50)	0.715/1.000	n.a	n.a	n.a	n.a	n.a	n.a
D	G/G	307 (0.501)	138 (0.437)	1.00		n.a	n.a	n.a	n.a	n.a	n.a
	G/T-T/T	306 (0.499)	178 (0.563)	0.77 (0.59–1.01)	0.060/0.672	n.a	n.a	n.a	n.a	n.a	n.a
R	G/G-G/T	556 (0.907)	288 (0.911)	1.00		n.a	n.a	n.a	n.a	n.a	n.a
	T/T	57 (0.093)	28 (0.089)	1.05 (0.66–1.69)	0.829/0.873	n.a	n.a	n.a	n.a	n.a	n.a
rs7041											
CD	C/C	185 (0.301)	69 (0.22)	1.00		n.a	n.a	n.a	n.a	n.a	n.a
	C/A	299 (0.487)	166 (0.53)	0.67 (0.48–0.94)	**0.020/0.590**	n.a	n.a	n.a	n.a	n.a	n.a
	A/A	130 (0.212)	78 (0.249)	0.62 (0.42–0.92)	**0.017/0.590**	n.a	n.a	n.a	n.a	n.a	n.a
D	C/C	185 (0.301)	69 (0.22)	1.00		n.a	n.a	n.a	n.a	n.a	n.a
	C/A-A/A	429 (0.699)	244 (0.78)	0.65 (0.48–0.90)	**0.008/0.355**	n.a	n.a	n.a	n.a	n.a	n.a
R	C/C-C/A	484 (0.788)	235 (0.751)	1.00		n.a	n.a	n.a	n.a	n.a	n.a
	A/A	130 (0.212)	78 (0.249)	0.81 (0.59–1.11)	0.192/0.707	n.a	n.a	n.a	n.a	n.a	n.a
*IL4*											
rs35648164											
CD	C/C	470 (0.992)	333 (0.971)	1.00		427 (0.986)	1.00		760 (0.979)	1.00	
	C/T	4 (0.008)	10 (0.029)	0.29 (0.09–0.93)	**0.037/0.693**	6 (0.014)	0.60 (0.17–2.16)	0.439/1.000	16 (0.021)	0.41 (0.13–1.22)	0.108/0.634
*RXRB*											
rs6531											
CD	A/A	267 (0.559)	221 (0.61)	1.00		260 (0.59)	1.00		481 (0.599)	1.00	
	A/G	184 (0.385)	112 (0.309)	1.35 (1.00–1.81)	**0.047/0.693**	158 (0.358)	1.14 (0.87–1.50)	0.351/1.000	270 (0.336)	1.23 (0.97–1.56)	0.093/0.616
	G/G	27 (0.057)	29 (0.08)	0.77 (0.44–1.34)	0.358/0.886	23 (0.052)	1.15 (0.64–2.06)	0.640/1.000	52 (0.065)	0.94 (0.57–1.52)	0.789/0.984
	A/A	267 (0.559)	221 (0.61)	1.00		260 (0.59)	1.00		481 (0.599)	1.00	
	A/G-G/G	211 (0.441)	141 (0.39)	1.23 (0.93–1.63)	0.144/0.694	181 (0.41)	1.14 (0.88–1.48)	0.327/0.813	322 (0.401)	1.18 (0.94–1.48)	0.156/0.655
R	A/A-A/G	451 (0.943)	333 (0.92)	1.00		418 (0.948)	1.00		751 (0.935)	1.00	
	G/G	27 (0.057)	29 (0.08)	0.69 (0.40–1.19)	0.181/0.707	23 (0.052)	1.09 (0.62–1.93)	0.763/0.993	52 (0.065)	0.86 (0.54–1.40)	0.549/0.821
*VDR*											
rs2228570											
CD	G/G	218 (0.365)	142 (0.408)	1.00		189 (0.451)	1.00		331 (0.432)	1.00	
	G/A	296 (0.495)	159 (0.457)	1.21 (0.91–1.61)	0.193/0.839	184 (0.439)	1.40 (1.07–1.82)	**0.015/0.326**	343 (0.447)	1.31 (1.04–1.65)	0.022/0.475
	A/A	84 (0.141)	47 (0.135)	1.17 (0.77–1.77)	0.467/0.932	46 (0.11)	1.57 (1.04–2.36)	**0.031/0.539**	93 (0.121)	1.37 (0.97–1.93)	0.070/0.616
D	G/G	218 (0.365)	142 (0.408)	1.00		189 (0.451)	1.00		331 (0.432)	1.00	
	G/A-A/A	380 (0.635)	206 (0.592)	1.20 (0.91–1.57)	0.189/0.694	230 (0.549)	1.43 (1.11–1.84)	**0.006/0.126**	436 (0.568)	1.32 (1.06–1.65)	**0.012/0.220**
R	G/G-G/A	514 (0.86)	301 (0.865)	1.00		373 (0.89)	1.00		674 (0.879)	1.00	
	A/A	84 (0.141)	47 (0.135)	1.05 (0.72–1.54)	0.801/0.870	46 (0.11)	1.31 (0.89–1.93)	0.162/0.993	93 (0.121)	1.18 (0.86–1.62)	0.298/0.728
*CYP24A1*											
rs2762934											
CD	G/G	169 (0.554)	170 (0.55)	1.00		295 (0.632)	1.00		465 (0.599)	1.00	
	G/A	119 (0.39)	112 (0.362)	1.06 (0.75–1.48)	0.753/1.000	142 (0.304)	1.46 (1.08–1.99)	**0.015/0.326**	254 (0.327)	1.29 (0.97–1.70)	0.077/0.616
	A/A	17 (0.056)	27 (0.087)	0.63 (0.33–1.20)	0.158/0.839	30 (0.064)	0.99 (0.53–1.85)	0.972/1.000	57 (0.073)	0.82 (0.46–1.45)	0.498/0.894
D	G/G	169 (0.554)	170 (0.55)	1.00		295 (0.632)	1.00		465 (0.599)	1.00	
	G/A-A/A	136 (0.446)	139 (0.45)	0.97 (0.71–1.34)	0.863/0.994	172 (0.368)	1.38 (1.03–1.85)	**0.031/0.326**	311 (0.401)	1.20 (0.92–1.57)	0.178/0.655
R	G/G-G/A	288 (0.944)	282 (0.913)	1.00		437 (0.936)	1.00		719 (0.927)	1.00	
	A/A	17 (0.056)	27 (0.087)	0.61 (0.33–1.15)	0.125/0.707	30 (0.064)	0.86 (0.47–1.59)	0.626/0.993	57 (0.073)	0.75 (0.43–1.30)	0.292/0.728

BH:Benjamini–Hochberg correction.

*Some SNVs, could not be determined in the group of controls due to DNA, shortage these are labelled in the table as not available (n.a.).

In addition, CR NSAIDs HRs patients were classified according to their symptoms. Our findings showed that the SNVs *GC* rs139523630, *IL4R* rs1049631, and *CYP24A1* rs2762934 were associated with a lower risk of developing isolated cutaneous symptoms in CR NSAIDs hypersensitivity patients (Supplementary Table S6). Nonetheless, following correction for multiple testing, none of the SNVs remained statistically significant. Moreover, those CR NSAIDs HRs patients carrying the FCER1G rs11421 mutant allele in homozygosis showed a higher risk of presenting anaphylaxis (Supplementary Table S7).

### 3.5 Differential effects in SNIUAA and CR patients

Certain SNVs have demonstrated a common association with both categories of NSAIDs HRs: SNIUAA and CR NSAIDs HRs. To determine those SNVs that may be specifically associated with each category of NSAID HRs, we compared the genotypic frequencies of these twenty-six SNVs among the SNIUAA and CR NSAIDs HRs group. The results of this analysis are shown in Supplementary Table S8. None of these SNVs remained statistically associated after adjustment for multiple comparisons.

### 3.6 Genetic association of serum IgE and vitamin D levels among NSAIDs hypersensitivity patients

**FIGURE 1:**
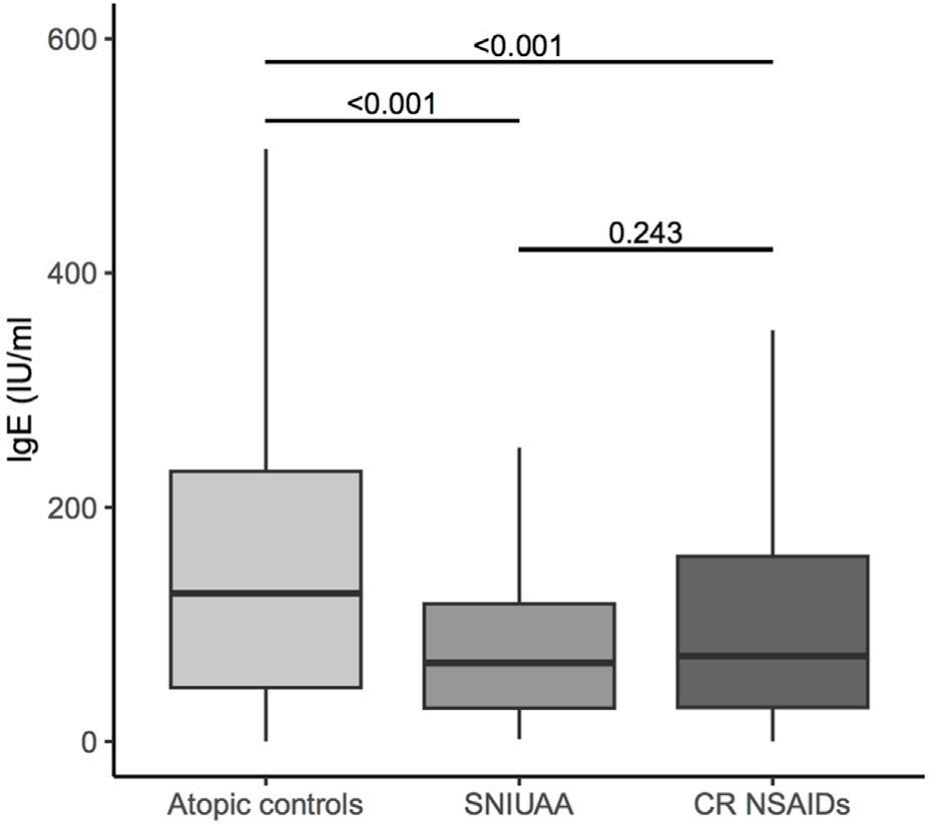
Box-plots of serum IgE levels by study group (pairwise comparison p-values obtained from Dunn’s test with Bonferroni correction).

Firstly, we compared the serum IgE levels of atopic-tolerant individuals and NSAIDs hypersensitivity patients. The global analysis indicates significant differences between study groups (*P*-value <0.001). Additionally, pairwise comparisons revealed higher serum IgE levels in atopic tolerant individuals (mean equal to 254.5 IU/mL) than in CR NSAIDs HRs (mean equal to 174.0 IU/mL) and SNIUAA (mean equal to 143.8 IU/mL), these differences being statistically significant (Figure 1). Besides, statistically significant differences in the serum IgE levels between these two categories of NSAIDs HRs were not observed (*P*-value = 0.243).

Additionally, these patients were stratified according to several clinical symptoms and demographic data to determine the association of these characteristics with the serum IgE levels of NSAIDs HRs patients (Table 6). Focusing on CR NSAIDs HRs patients, we observed that women showed lower serum IgE levels than men (*P*-value: 0.0004). Also, CR NSAIDs HRs patients with respiratory symptoms demonstrated significantly lower serum IgE levels than CR NSAIDs HRs patients without respiratory symptoms (*P*-value: 0.031). Concerning SNIUAA patients, those with isolated cutaneous symptoms which include urticaria and/or angioedema demonstrated significantly lower serum IgE levels than those patients without those symptoms (*P*-value: 0.012). Also, patients with a history of atopy showed higher serum IgE levels (*P*-value: 0.002) (Table 6).

**TABLE 6:** Association of serum IgE levels with demographic and clinical data of individuals included in the study.

Variables		N, Mean ± standard deviation (µ ± σ) (IU/mL), p- value (Kruskal–Wallis)								
		Atopic controls			SNIUAA			CR NSAIDs		
		N	(µ ± σ)	p- value	N	(µ ± σ)	p- value	N	(µ ± σ)	p- value
Sex	M	179	263.74 ± 340.88	0.147	36	231.7 ± 647.3	0.134	171	221.8 ± 343.4	**0.0004**
	F	219	252.64 ± 449.03		74	101.0 ± 130.6		238	139.1 ± 301.5	
History of atopy	No	0	-	-	77	126.5 ± 441.4	**0.002**	310	173.5 ± 333.1	0.087
	Yes	398	253.83 ± 401.60		33	184.1 ± 211.9		99	175.6 ± 286.4	
History of urticaria	No	-	-	-	108	144.6 ± 390.4	0.737	389	171.4 ± 312.3	0.246
	Yes	-	-		2	99.5 ± 75.7		3	41.1 ± 14.8	
Anaphylaxis	No	-	-	-	71	121.6 ± 136.7	0.095	380	155.1 ± 220.9	0.435
	Yes	-	-		37	187.7 ± 642.9		27	450.0 ± 911.4	
Cutaneous	No	-	-	-	58	153.9 ± 518.7	**0.012**	86	251.1 ± 561.0	0.610
	Yes	-	-		50	133.0 ± 139.2		321	154.2 ± 215.8	
Respiratory	No	-	-	-	106	142.9 ± 393.8	0.072	388	179.1 ± 329.3	**0.031**
	Yes	-	-		2	213.5 ± 67.2		19	84.6 ± 97.4	
Blended	No	-	-	-	103	148.7 ± 399.0	0.270	375	170.5 ± 323.3	0.537
	Yes	-	-		5	51.6 ± 49.7		32	223.7 ± 316.8	
Other symptoms	No	-	-	-	94	151.9 ± 414.4	0.163	399	176.5 ± 325.6	0.419
	Yes	-	-		14	92.8 ± 147.9		8	85.4 ± 69.6	

M = Male; F=Female.

Blended reactions: cutaneous and respiratory symptoms.

Others: exanthema, erythema, eczema, glottic edema, rash or pruritus.

To analyze the genetic associations with serum IgE levels, generalized linear models adjusted by sex were carried out (Table 7). After adjustment for multiple comparisons according to each genetic model, one variant remained statistically associated with serum IgE levels (*P*-value: 0.021. Thus, CR NSAIDs HRs patients who were homozygous for the mutant allele of the SNV *RXRB* rs2744537 exhibited higher serum IgE levels. Concerning SNIUAA patients, none of the analyzed SNVs were significantly associated with serum IgE levels. However, the haplotype conformed by the SNVs *RXRB* rs2744537-rs6531-6:33161660 (A-G-T) showed a significant association with increased serum IgE levels in CR NSAIDs hypersensitivity patients (*P*-value <0.0001); as well as in SNIUAA patients (*P*-value <0.0001).

**TABLE 7:** SNVs statistically significant associated with serum IgE levels (adjusted by sex).

	All subjects		Atopic controls		SNIUAA		CR NSAIDs	
Gene_SNV	IgE (mean ± SD)	p-value (GLM)/Bonf	IgE (mean ± SD)	p-value (GLM)/Bonf	IgE (mean ± SD)	p-value (GLM)/Bonf	IgE (mean ± SD)	p-value (GLM)/Bonf
*RXRB*								
rs2744537								
A/A	212.46 ± 368.25		258.27 ± 457.45		142.21 ± 175.04		178.35 ± 271.63	
A/C	190.67 ± 338.07	0.384/1.000	249.25 ± 336.08	0.932/1.000	167.12 ± 600.59	0.805/1.000	136.22 ± 191.09	0.054/0.162
C/C	298.25 ± 375.23	0.115/0.345	353.40 ± 290.23	0.335/1.000	39.00 ± 32.58	0.602/1.000	333.84 ± 476.47	0.007/0.021
rs6531								
A/A	200.09 ± 297.27		234.38 ± 338.73		138.48 ± 171.61		178.10 ± 269.72	
A/G	185.29 ± 318.05	0.473/1.000	233.65 ± 292.31	0.922/1.000	172.40 ± 607.66	0.800/1.000	137.52 ± 191.16	0.051/0.153
G/G	366.18 ± 737.07	**0.001/0.004**	551.95 ± 1.013.92	**0.000381/0.001**	40.83 ± 35.30	0.618/1.000	281.43 ± 453.69	0.069/0.207

GLM: generalized linear model; Bonf: Bonferroni correction.

We also analyzed serum vitamin D levels in patients with NSAID hypersensitivity reactions (NSAIDs HRs). Results are summarized in Supplementary Figure S1 and Supplementary Tables S9, S10. Significant differences were found between groups (P < 0.001), with non-atopic NSAIDs-tolerant individuals showing the lowest levels (mean: 18.6 ng/mL). All groups had levels below the recommended 30–50 ng/mL range. Patients with blended reactions had significantly higher vitamin D levels (P = 0.002), while those with a history of atopy had lower levels (P = 0.003), particularly in the CR NSAIDs HRs group.

Genetic analysis revealed that certain SNVs (GC rs4588, GC rs7041, IL4R rs1805010) were associated with lower vitamin D levels in non-atopic tolerant patients. In SNIUAA patients, IL4R rs3024678 was significantly linked to higher vitamin D levels (P = 0.0002; corrected P = 0.002).

## 4 Discussion

This study aimed to investigate the genetic and immunological factors underlying the mechanisms of NSAID hypersensitivity reactions (HRs), with particular emphasis on the high-affinity IgE receptor and genes involved in the vitamin D pathway. Our findings have identified 216 variants including 186 not previously reported in genes *FCER1A, MS4A2, FCER1G, VDR, GC, CYP2R1, CYP27A1, CYP27B1, CYP24A1, RXRA, RXRB, RXRG, IL4, IL4R, IL13*, and *IL13RA1*. Notably, SNVs in *VDR* and *CYP24A1* were linked to SNIUAA susceptibility, while *FCER1G* rs11421 was associated with a higher risk of anaphylaxis in CR NSAIDs hypersensitivity patients. Additionally, a haplotype comprising SNVs in the *RXRB* gene was significantly associated with elevated serum IgE level in SNIUAA and CR NSAIDs hypersensitivity patients. These findings suggest that both immunological and non-immunological mechanisms may contribute to the pathophysiology of NSAID HRs.

Genetic variants constitute one of the main sources of variability in the response to drugs (Naisbitt et al., 2003; Kuruvilla et al., 2022). Most genetic association studies follow a case-control design, genotyping a small set of polymorphisms, or genome-wide association studies (GWAS) (Zo et al., 2007). Both approaches have shown ineffectiveness in detecting rare variants or have demonstrated false-positive associations with a lack of functional effects (Boyle et al., 2017; Ziegler and Sun, 2012). NGS techniques have provided the capability to cover larger DNA areas thus detecting novel SNVs and analyzing variants not previously included in association studies (Amo et al., 2019). Regarding NSAIDs HRs, CR mediated by non-specific immunological mechanisms and SR whose mechanism is attributed to specific IgE antibodies or T-cells, may be distinguished (Kowalski et al., 2013).

Concerning the analysis of individual SNVs, the most relevant variants associated were generally weak. However, these genetic variants demonstrated a differential pattern of associations according to the underlying mechanism of these HDRs. Therefore, further validation in larger, ethnically diverse cohorts, along with mechanistic studies, is essential to confirm these findings and evaluate their applicability in pharmacogenetics clinical decision support systems. Furthermore, the analysis of serum IgE levels in NSAIDs HRs demonstrated non-statistically significant differences between SNIUAA and CR NSAIDs HRs reactions. By turn, we identified statistically significant associations between SNVs in *RXRB* gene and serum IgE levels in CR NSAIDs hypersensitivity patients. Nevertheless, *RXRB* rs2744537 (3’UTR) was the only variant that remained significant after adjustment for multiple comparisons. RXR is a family of nuclear hormone receptors that regulate gene expression by binding to specific DNA sequences in target genes. There are three isoforms: RXRα, RXRβ and RXRγ. Among them, the isoform RXRβ, which is encoded by the gene *RXRB*, is expressed in a wide range of tissues (Bastien and Rochette-Egly, 2004). Several studies have highlighted the role of RXRs in coordinating gene expression related to immunity and inflammation (Núñez et al., 2010; Roszer et al., 2013). However, the functional impact of the SNV *RXRB* rs2744537 remains poorly understood. Our findings suggest a potential crosstalk between RXRB signaling and serum IgE production. Further functional studies are needed to clarify the role of RXR in the mechanisms underlying hypersensitivity drug reactions. Our study shows low levels of vitamin D in the analyzed population since the mean vitamin D levels did not reach the recommended levels (Varsavsky et al., 2017). Our findings suggest that the risk of developing CR to NSAIDs may be influenced by a history of atopy (*P*-value <0.001), and low levels of vitamin D (*P*-value = 0.008). Regarding the influence of the SNVs analyzed on serum vitamin D levels, the mutant alleles of the nonsynonymous SNVs *GC* rs4588 (Thr436Lys) and rs7041 (Asp432Glu) were associated with lower vitamin D levels in non-atopic tolerant individuals as has been previously reported (Supplementary Material) (Rozmus et al., 2022). Nevertheless, these associations were not found in CR or SNIUAA HRs patients (Varsavsky et al., 2017) (Supplementary Material, Supplementary Table S10). However, the serum vitamin D levels observed across all NSAIDs hypersensitivity subgroups, as well as in the control cohorts, were consistently below the thresholds recommended for the general population. Additionally, recent research based on prospective cohort studies has reported that serum 25-(OH) vitamin D above 30 ng/mL reduces the risk of cardiovascular events and autoimmune diseases whereas lower concentrations are qualified as vitamin D deficiency (Grant et al., 2025). In addition, given the impact of solar radiation on vitamin D synthesis, a limitation of our study is the collection of serum samples across different seasons. Accordingly, the vitamin D levels measured correspond to the baseline status of the individuals at the time of sampling. Therefore, determining whether vitamin D plays a significant role in HRs to NSAIDs and in the regulation of IgE synthesis through some of its effectors, such as IL4 and IL13, or through the effect of the metabolite itself 1,25(OH)_2_D_3,_ or through the VDR receptor (Chen et al., 2007) requires further investigation to be fully elucidated. This study has limitations that should be considered when interpreting the findings. First, although some associations lost statistical significance after correction for multiple comparisons, the use of Bonferroni correction enhances the reliability of the results. Second, the study population consisted exclusively of individuals of Caucasian Spanish origin. While this homogeneity strengthens internal validity by minimizing genetic background variability, it may limit the generalizability of the findings to other ethnic groups. However, the large sample size and consistent methodology across groups help mitigate this limitation. Third, serum vitamin D levels were measured at a single time point without controlling for seasonal variation, which is known to influence vitamin D status due to differences in sun exposure. Finally, although the study identified several promising genetic associations, functional validation and replication in independent, ethnically diverse cohorts are necessary to confirm their clinical relevance. Such limitations are typical of complex genetic studies and underscore key areas for future research.

## 5 Conclusion

Our findings underscore the relevance of genetic and serum biomarkers particularly SNVs in vitamin D and IgE-related pathways in enhancing the understanding, diagnosis, and potential stratification of NSAIDs HRs.

## Data Availability

The original contributions presented in the study are publicly available. This data can be found here: Institutional repository DEHESA of University of Extremadura: https://dehesa.unex.es/bitstreams/3298d4b0-be12-4f90-bf02-7178fdad4d1d/download.
