# Supplementary material for: Genetic and serum biomarkers of NSAID hypersensitivity reactions
Source: Front Pharmacol. 2025 Oct 2;16:1502755. doi: 10.3389/fphar.2025.1502755 (PMC12528179; doi:10.3389/fphar.2025.1502755)
Supplement: Supplementary file 1 [file DataSheet1.docx]

Supplementary material

We aimed to analyze the vitamin D levels in NSAIDs hypersensitivity reactions patients. Our findings showed significant differences between groups (*P*-value < 0.001). According to pairwise comparisons, non-atopic NSAIDs-tolerant individuals exhibited lower levels of serum vitamin D (mean equal to 18.6 ng/ml). This difference was statistically significant in comparison with SNIUAA patients (mean equal to 25.2 ng/ml), CR NSAIDs hypersensitivity patients (mean equal to 22.1 ng/ml), and atopic NSAIDs tolerant individuals (mean equal to 23.9 ng/ml) (Fig S1). In all the analyzed groups, the serum vitamin D was found lower than the range of 30-50 ng/mL which is recommended to achieve the health benefits provided by vitamin D (1). Patients were stratified according to different clinical symptoms and demographic data to determine the influence of these characteristics on the serum vitamin D levels of NSAIDs HRs patients (Table S9). The analysis demonstrated that sex is not a determining factor, nor the history of urticaria or the clinical presentation, except for patients who presented blended reactions. These patients with blended symptoms demonstrated significantly higher serum vitamin D levels than those patients without blended symptoms (*P*-value: 0.002). Also, patients with a history of atopy showed statistically significantly lower serum vitamin D levels (*P*-value: 0.003); this difference being attributable to the group of CR NSAIDs HRs patients. In contrast, non-atopic controls exhibited lower serum vitamin D levels compared to the atopic control group.

To analyze the association between the SNVs selected and serum vitamin D levels, generalized linear models adjusted by sex were carried out (Table S10). It was observed that non-atopic tolerant patients carrying the *GC* rs4588, *GC* rs7041 or *IL4R* rs1805010 mutant alleles showed lower vitamin D levels. Nevertheless, none of the SNVs analyzed were significantly associated with the serum vitamin D levels in CR NSAIDs hypersensitivity patients. Concerning SNIUAA patients, the SNV *IL4R* rs3024678 (*P*-value: 0.0002; *P*-value corrected: 0.002) was significantly associated with higher serum vitamin D levels.

1. Varsavsky M, Rozas Moreno P, Becerra Fernández A, Luque Fernández I, Quesada Gómez JM, Ávila Rubio V, et al. Recommended vitamin D levels in the general population. Endocrinol Diabetes Nutr. 2017;64:7–14.

Table S1. Characteristics of the selected individuals included in the identification of genetic variantsusing next-generation sequencing.

|  | HEALTHY TOLERANT CONTROLS  (n=22) | ATOPIC TOLERANT CONTROLS  (n=22) | SNIUAA  (n=41) | CR NSAIDs  (n=46) | p-value |
| --- | --- | --- | --- | --- | --- |
| Women, n (%) | 14 (63.6%) | 8 (36.4%) | 26 (63.4%) | 29 (63%) | 0.140 |
| Age + SD (range) | 23.9 ± 8.8 (20-54) | 30 ± 12.5 (14-58) | 43.2 ± 15.7 (5-77) | 45.3 ± 14.2 (20-79) | <0.001 |
| History of atopy (N,%) | 0 | 22 (100%) | 14 (34.1%) | 9 (19.6%) | n.a |

n.a: no**n** applicable.

**Table S2**: Details of the sequenced areas (GRCh37 assembly of the human genome)

| Coordinate origin | Coordinate end | Coverage |
| --- | --- | --- |
| chr1:159258504 | chr1:159259543 | 100.00 % |
| chr1:159272096 | chr1: 159272209 | 100.00 % |
| chr1:159272644 | chr1:159272664 | 100.00 % |
| chr1:159273718 | chr1:159273972 | 100.00 % |
| chr1:159275778 | chr1: 159276035 | 100.00 % |
| chr1:161184111 | chr1:161185160 | 100.00 % |
| chr1:165379939 | chr1: 165380068 | 100.00 % |
| chr1:165380186 | chr1:165380346 | 100.00 % |
| chr1:165386278 | chr1:165386457 | 100.00 % |
| chr1:165389107 | chr1:165389253 | 100.00 % |
| chr1:165393985 | chr1:165394209 | 100.00 % |
| chr1:165397954 | chr1:165398203 | 100.00 % |
| chr1:165406161 | chr1:165406441 | 100.00 % |
| chr1:165414080 | chr1:165415430 | 100.00 % |
| chr2:219645905 | chr2:219647160 | 100.00 % |
| chr2:219674300 | chr2:219674490 | 100.00 % |
| chr2:219676945 | chr2:219677819 | 100.00 % |
| chr2:219678744 | chr2:219679480 | 100.00 % |
| chr4:72631149 | chr4:72631360 | 100.00 % |
| chr4:72634018 | chr4:72634150 | 100.00 % |
| chr4:72635048 | chr4:72635319 | 100.00 % |
| chr4:72649678 | chr4:72650079 | 100.00 % |
| chr4:72669643 | chr4:72670664 | 91.62 % |
| chr5:131991955 | chr5:131992323 | 100.00 % |
| chr5:131992756 | chr5:131992937 | 100.00 % |
| chr5:131993864 | chr5:131995163 | 100.00 % |
| chr5:131995416 | chr5:131995520 | 100.00 % |
| chr5:131995867 | chr5:131996802 | 100.00 % |
| chr5:132008742 | chr5:132009877 | 100.00 % |
| chr5:132010049 | chr5:132010198 | 100.00 % |
| chr5:132015406 | chr5:132015582 | 100.00 % |
| chr5:132018178 | chr5:132019280 | 100.00 % |
| chr6:33161365 | chr6:33162606 | 100.00 % |
| chr6:33162722 | chr6:33163243 | 100.00 % |
| chr6:33163347 | chr6:33163479 | 100.00 % |
| chr6:33163561 | chr6:33164383 | 100.00 % |
| chr6:33165539 | chr6:33165718 | 100.00 % |
| chr6:33166085 | chr6:33166414 | 100.00 % |
| chr6:33166946 | chr6:33167193 | 100.00 % |
| chr6:33168019 | chr6:33169254 | 100.00 % |
| chr9:137208940 | chr9:137211350 | 99.59 % |
| chr9:137217900 | chr9:137218505 | 100.00 % |
| chr9:137328313 | chr9:137332431 | 100.00 % |
| chr11:14898550 | chr11:14900989 | 100.00 % |
| chr11:14901682 | chr11:14902314 | 100.00 % |
| chr11:14907322 | chr11:14908882 | 100.00 % |
| chr11:14911978 | chr11:14913208 | 97.48 % |
| chr11:14913527 | chr11:14914751 | 100.00 % |
| chr11:59855238 | chr11:59856294 | 100.00 % |
| chr11:59857165 | chr11:59857294 | 100.00 % |
| chr11:59857809 | chr11:59857943 | 100.00 % |
| chr11:59860265 | chr11:59860321 | 100.00 % |
| chr11:59860873 | chr11:59861031 | 100.00 % |
| chr11:59861437 | chr11:59861535 | 100.00 % |
| chr11:59863031 | chr11:59863800 | 100.00 % |
| chr12:48235300 | chr12:48238788 | 99.71 % |
| chr12:48240118 | chr12:48240234 | 100.00 % |
| chr12:48240440 | chr12:48240591 | 100.00 % |
| chr12:48249413 | chr12:48249584 | 100.00 % |
| chr12:48250912 | chr12:48251032 | 100.00 % |
| chr12:48251287 | chr12:48251471 | 100.00 % |
| chr12:48258830 | chr12:48258960 | 100.00 % |
| chr12:48272599 | chr12:48272897 | 100.00 % |
| chr12:48276476 | chr12:48276719 | 100.00 % |
| chr12:48293619 | chr12:48293742 | 100.00 % |
| chr12:48298340 | chr12:48299414 | 100.00 % |
| chr12:48336470 | chr12:48337000 | 100.00 % |
| chr12:58156000 | chr12:58157038 | 100.00 % |
| chr12:58157394 | chr12:58157591 | 100.00 % |
| chr12:58157881 | chr12:58157959 | 100.00 % |
| chr12:58158161 | chr12:58158333 | 100.00 % |
| chr12:58158537 | chr12:58158709 | 100.00 % |
| chr12:58158794 | chr12:58159466 | 100.00 % |
| chr12:58159790 | chr12:58160295 | 100.00 % |
| chr12:58160630 | chr12:58161825 | 99.25 % |
| chr16:27324989 | chr16:27325562 | 100.00 % |
| chr16:27326712 | chr16:27326887 | 100.00 % |
| chr16:27329955 | chr16:27330089 | 100.00 % |
| chr16:27341386 | chr16:27341520 | 100.00 % |
| chr16:27345637 | chr16:27345739 | 100.00 % |
| chr16:27351502 | chr16:27351596 | 100.00 % |
| chr16:27351609 | chr16:27352634 | 100.00 % |
| chr16:27353350 | chr16:27353581 | 100.00 % |
| chr16:27356188 | chr16:27356412 | 100.00 % |
| chr16:27356504 | chr16:27356925 | 100.00 % |
| chr16:27357786 | chr16:27357939 | 100.00 % |
| chr16:27363861 | chr16:27364017 | 100.00 % |
| chr16:27365404 | chr16:27365484 | 100.00 % |
| chr16:27366339 | chr16:27366533 | 100.00 % |
| chr16:27366667 | chr16:27367231 | 100.00 % |
| chr16:27370237 | chr16:27370315 | 100.00 % |
| chr16:27372087 | chr16:27372136 | 100.00 % |
| chr16:27372259 | chr16:27372342 | 100.00 % |
| chr16:27373573 | chr16:27376500 | 100.00 % |
| chr20:52769977 | chr20:52771491 | 99.60 % |
| chr20:52773707 | chr20:52773837 | 97.71 % |
| chr20:52773927 | chr20:52774124 | 100.00 % |
| chr20:52774625 | chr20:52774703 | 100.00 % |
| chr20:52775496 | chr20:52775662 | 100.00 % |
| chr20:52779256 | chr20:52779401 | 100.00 % |
| chr20:52780991 | chr20:52781102 | 100.00 % |
| chr20:52782281 | chr20:52782372 | 100.00 % |
| chr20:52786131 | chr20:52786227 | 96.91 % |
| chr20:52788116 | chr20:52788492 | 100.00 % |
| chr20:52789339 | chr20:52789638 | 100.00 % |
| chr20:52789861 | chr20:52791119 | 100.00 % |
| chrx:117860601 | chrx:117861689 | 100.00 % |
| chrx:117874980 | chrx:117875119 | 100.00 % |
| chrx:117880917 | chrx:117881055 | 100.00 % |
| chrx:117883621 | chrx:117883741 | 100.00 % |
| chrx:117892018 | chrx:117892205 | 100.00 % |
| chrx:117892827 | chrx:117892932 | 100.00 % |
| chrx:117895101 | chrx:117895485 | 100.00 % |
| chrx:117900493 | chrx:117900540 | 100.00 % |
| chrx:117900807 | chrx:117900939 | 100.00 % |
| chrx:117903616 | chrx:117904633 | 100.00 % |
| chrx:117907842 | chrx:117907938 | 100.00 % |
| chrx:117910390 | chrx:117910474 | 100.00 % |
| chrx:117925725 | chrx:117928490 | 100.00 % |

Table S3. Genetic variants identified in the sequencing study.

|  |  |  |  | **TOTAL**  **(N=131)** | **High IgE levels**  **(N=10)** | **High VitD levels (N=10)** | **Low VitD levels (N=10)** | **SNIUA vs Non-atopic Controls** | **SNIUAA vs Atopic Controls** | **SNIUAA vs Controls** | **CR vs non-atopic controls** | **CR vs Atopic controls** | **CR vs controls** |  |
| --- | --- | --- | --- | --- | --- | --- | --- | --- | --- | --- | --- | --- | --- | --- |
| **Gene/SNP** | **Chromosomal Location** | **Alleles** | **Consequence** | **MAF** | **MAF** | **MAF** | **MAF** | **p-value** | **p-value** | **p-value** | **p-value** | **p-value** | **p-value** | **Previously reported** |
| ***FCER1A*** |  |  |  |  |  |  |  |  |  |  |  |  |  |  |
| **rs200728340** | 1:159272209 | G/A | Ala19Thr | 0.004 | 0 | 0 | 0 | 0.13 | -- | 0.19 | 0.14 | -- | 0.21 | NO |
| **rs201314370** | 1:159273791 | A/G | Thr50 | 0.004 | 0 | 0 | 0 | 0.37 | 0.27 | 0.19 | -- | -- | -- | NO |
| **rs2298805** | 1:159273943 | G/A | Ser101Asn | 0.004 | 0 | 0.050 | 0 | 0.37 | 0.27 | 0.19 | -- | -- | -- | YES |
| **rs41264475** | 1:159277689 | C/A | Asn247Lys | 0.034 | 0 | 0.050 | 0.050 | 0.96 | 0.82 | 0.91 | 0.25 | 0.32 | 0.21 | NO |
| **rs143419433** | 1:159277709 | C/T | Pro254Ser | 0.004 | 0 | 0 | 0 | -- | 0.2 | 0.28 | -- | 0.22 | 0.30 | NO |
| **rs7549785** | 1:159277868 | G/A | 3´UTR | 0.144 | 0.150 | 0.100 | 0.200 | 0.86 | 0.4 | 0.8 | 0.29 | 0.53 | 0.58 | NO |
| ***FCER1G*** |  |  |  |  |  |  |  |  |  |  |  |  |  |  |
| **rs3557** | 1:161188893 | T/G | 3´UTR | 0.088 | 0.100 | 0.100 | 0.100 | 0.45 | 0.88 | 0.52 | 0.58 | 0.62 | 0.52 | NO |
| **rs11421** | 1:161188936 | T/C | 3´UTR | 0.178 | 0.200 | 0.250 | 0.250 | 0.29 | 0.81 | 0.58 | 0.57 | 0.44 | 0.58 | NO |
| **161189009** | 1:161189009 | C/T | 3´UTR | 0.004 | 0.050 | 0 | 0 | -- | 0.20 | 0.28 | -- | 0.22 | 0.30 | NO |
| **rs188894565** | 1:161189034 | G/T | 3´UTR | 0.008 | 0 | 0 | 0 | -- | -- | -- | 0.19 | 0.17 | 0.08 | NO |
| **161189067** | 1:161189067 | AT/A | 3’ UTR | 0.004 | 0 | 0 | 0 | -- | -- | -- | 0.35 | 0.46 | 0.26 | NO |
| **rs4634925** | 1:161189344 | G/C | 3’ UTR | 0.361 | 0.300 | 0.350 | 0.450 | 0.07 | 0.89 | 0.19 | 0.10 | 0.44 | 0.16 | NO |
| **rs41270851** | 1:161189391 | A/G | 3’ UTR | 0.011 | 0 | 0 | 0 | 0.21 | 0.88 | 0.50 | -- | 0.22 | 0.30 | NO |
| **rs115159907** | 1:161189733 | A/C | 3’ UTR | 0.015 | 0 | 0.050 | 0 | 0.97 | 0.88 | 0.90 | 0.14 | 0.22 | 0.14 | NO |
| **rs2502802** | 1:161189812 | T/C | 3’ UTR | 0.012 | 0.050 | 0 | 0 | 0.12 | 0.08 | 0.09 | 0.64 | 0.67 | 0.67 | NO |
| **rs7528588** | 1:161189934 | C/G | 3’ UTR | 0.178 | 0.200 | 0.250 | 0.250 | 0.29 | 0.81 | 0.58 | 0.57 | 0.44 | 0.58 | YES |
| **rs189851377** | 1:161190089 | G/A | 3’ UTR | 0.004 | 0 | 0 | 0 | -- | -- | -- | 0.35 | 0.46 | 0.26 | NO |
| **rs12409855** | 1:161190182 | G/A | 3’ UTR | 0.004 | 0 | 0 | 0 | -- | -- | -- | 0.35 | 0.46 | 0.26 | NO |
| **rs4503368** | 1:161190250 | T/C | Leu90Pro | 0.431 | 0.278 | 0.222 | 0.556 | 0.06 | 0.87 | 0.22 | 0.12 | 0.42 | 0.18 | YES |
| **rs558396984** | 1:161190267 | C/A | 3’ UTR | 0.004 | 0 | 0 | 0.050 | -- | -- | -- | 0.35 | 0.24 | 0.17 | NO |
| **rs2502801** | 1:161190276 | C/G | 3’ UTR | 0.292 | 0.200 | 0.444 | 0.111 | 0.28 | 0.39 | 0.44 | 0.80 | 0.56 | 0.98 | NO |
| **rs4348741** | 1:161190284 | A/C | 3’ UTR | 0.385 | 0.500 | 0.333 | 0.167 | 0.03 | 0.13 | 0.19 | 0.07 | 0.06 | 0.10 | NO |
| **rs116415980** | 1:161190301 | G/A | 3’ UTR | 0.004 | 0.050 | 0 | 0 | -- | 0.20 | 0.28 | -- | 0.22 | 0.30 | NO |
| **rs12408729** | 1:161190406 | A/G | 3’ UTR | 0.012 | 0 | 0 | 0 | -- | 0.48 | -- | 0.42 | 0.39 | 0.21 | NO |
| **rs146525792** | 1:161190435 | C/T | 3’ UTR | 0.007 | 0 | 0 | 0 | 0.37 | -- | 0.28 | -- | -- | -- | NO |
| ***RXRG*** |  |  |  |  |  |  |  |  |  |  |  |  |  |  |
| **rs189895016** | 1:165369627 | C/T | 3’ UTR | 0.007 | 0 | 0.050 | 0 | 0.2 | 0.32 | 0.13 | -- | -- | -- | NO |
| **rs59154518** | 1:165369858 | G/T | 3’ UTR | 0.078 | 0.100 | 0.100 | 0.056 | 0.69 | 0.48 | 0.53 | 0.24 | 0.82 | 0.64 | NO |
| **rs57548196** | 1:165369862 | T/C | 3’ UTR | 0.078 | 0.100 | 0.100 | 0.056 | 0.69 | 0.48 | 0.53 | 0.24 | 0.82 | 0.64 | NO |
| **rs145582845** | 1:165369869 | C/T | 3’ UTR | 0.007 | 0 | 0.050 | 0 | 0.37 | 0.61 | 0.99 | -- | 0.22 | 0.3 | NO |
| **165369957** | 1:165369957 | AG/A | 3’ UTR | 0.026 | 0 | 0.050 | 0 | 0.18 | 0.66 | 0.44 | 0.09 | 0.67 | 0.17 | NO |
| **rs78299594** | 1:165370148 | T/C | 3’ UTR | 0.049 | 0 | 0.200 | 0.100 | 0.47 | 0.10 | 0.17 | 0.81 | 0.62 | 0.98 | NO |
| **rs117775000** | 1:165370150 | C/T | 3’ UTR | 0.004 | 0 | 0 | 0 | -- | -- | -- | 0.35 | 0.46 | 0.26 | NO |
| **165370172** | 1:165370172 | CAT/C | 3´UTR | 0.023 | 0 | 0.050 | 0 | 0.44 | 0.66 | 0.77 | 0.25 | 0.67 | 0.34 | NO |
| **rs55901710** | 1:165370212 | G/A | 3´UTR | 0.219 | 0.300 | 0.150 | 0.250 | 0.64 | 0.27 | 0.62 | 0.46 | 0.67 | 0.53 | NO |
| **rs17429130** | 1:165370252 | G/C | 3´UTR | 0.034 | 0 | 0.050 | 0 | 0.54 | 0.41 | 0.99 | 0.09 | 0.67 | 0.17 | NO |
| **165370367** | 1:165370367 | T/TA | 3´UTR | 0.108 | 0.100 | 0.100 | 0.150 | 0.03 | 0.72 | 0.09 | 0.22 | 0.73 | 0.56 | NO |
| **rs56362364** | 1:165370400 | G/C | 3´UTR | 0.228 | 0.300 | 0.150 | 0.250 | 0.86 | 0.29 | 0.81 | 0.36 | 0.22 | 0.31 | NO |
| **rs10918169** | 1:165370475 | G/C | 3´UTR | 0.220 | 0.300 | 0.150 | 0.250 | 0.69 | 0.21 | 0.54 | 0.36 | 0.22 | 0.31 | NO |
| **rs2134095** | 1:165377552 | G/A | Val350 | 0.309 | 0.250 | 0.250 | 0.200 | 0.003 | 0.68 | 0.03 | 0.01 | 0.86 | 0.09 | NO |
| **rs769620178** | 1:165378881 | G/A | Ser320 | 0.004 | 0 | 0 | 0 | 0.13 | -- | 0.19 | 0.14 | -- | 0.21 | NO |
| **rs138711363** | 1:165378890 | G/A | Arg317 | 0.004 | 0 | 0.050 | 0 | 0.37 | 0.48 | 0.28 | -- | -- | -- | NO |
| **rs185905** | 1:165380027 | C/T | Lys275 | 0.023 | 0 | 0 | 0 | 0.02 | 0.89 | 0.09 | 0.003 | 0.22 | 0.01 | NO |
| **rs1128977** | 1:165389129 | G/A | Ala140 | 0.359 | 0.400 | 0.400 | 0.550 | 0.001 | 0.70 | 0.04 | 0.002 | 0.85 | 0.14 | NO |
| **rs113471** | 1:165398115 | T/C | Thr46 | 0.217 | 0.300 | 0.200 | 0.200 | 0.02 | 0.37 | 0.05 | 0.003 | 0.36 | 0.06 | NO |
| ***CYP27A1*** |  |  |  |  |  |  |  |  |  |  |  |  |  |  |
| **rs778193906** | 2:219646962 | C/T | Gly19 | 0.008 | 0 | 0 | 0 | 0.12 | -- | 0.28 | 0.14 | -- | 0.30 | NO |
| **rs757653354** | 2:219646967 | G/T | Cys21Tyr | 0.008 | 0 | 0 | 0 | 0.12 | -- | 0.28 | 0.14 | -- | 0.30 | NO |
| **rs765555645** | 2:219674428 | C/T | Asn128 | 0.004 | 0 | 0 | 0 | -- | -- | -- | 0.35 | 0.24 | 0.17 | NO |
| **rs692003** | 2:219674482 | G/A | Pro146 | 0.012 | 0 | 0 | 0.050 | 0.03 | -- | 0.09 | 0.25 | 0.46 | 0.56 | NO |
| **rs61733619** | 2:219677690 | A/G | Gln296 | 0.030 | 0 | 0 | 0 | 0.45 | 0.15 | 0.20 | 0.25 | 0.15 | 0.12 | NO |
| **rs139944377** | 2:219677761 | T/G | Leu320Arg | 0.011 | 0 | 0 | 0 | 0.10 | -- | 0.18 | 0.11 | -- | 0.20 | NO |
| **rs41272687** | 2:219678877 | C/T | Pro384Leu | 0.026 | 0 | 0.050 | 0.050 | 0.74 | 0.31 | 0.81 | 0.66 | 0.09 | 0.28 | NO |
| **rs72551323** | 2:219679475 | G/T | Ala491Ser | 0.008 | 0 | 0 | 0 | 0.13 | 0.08 | 0.06 | 0.14 | 0.08 | 0.07 | NO |
| **rs115360491** | 2:219680096 | G/T | 3’ UTR | 0.004 | 0 | 0 | 0 | -- | -- | -- | 0.35 | 0.24 | 0.17 | NO |
| **rs6730078** | 2:219680163 | C/T | 3’ UTR | 0.004 | 0 | 0 | 0 | -- | 0.08 | 0.19 | -- | 0.08 | 0.21 | NO |
| **rs151203082** | 2:219680290 | G/A | 3’ UTR | 0.011 | 0.050 | 0.050 | 0.050 | 0.12 | 0.08 | 0.03 | -- | -- | -- | NO |
| ***GC*** |  |  |  |  |  |  |  |  |  |  |  |  |  |  |
| **rs139523630** | 4:72606530 | T/C | 3’ UTR | 0.008 | 0 | 0 | 0.050 | -- | -- | -- | 0.18 | 0.10 | 0.05 | NO |
| **rs222048** | 4:72606587 | G/A | 3’ UTR | 0.008 | 0 | 0 | 0 | -- | 0.20 | 0.28 | -- | 0.22 | 0.30 | NO |
| **rs2070743** | 4:72606935 | G/A | 3’ UTR | 0.007 | 0 | 0 | 0 | 0.20 | 0.32 | 0.13 | -- | -- | -- | NO |
| **rs193201856** | 4:72607105 | A/G | 3’ UTR | 0.007 | 0 | 0 | 0 | 0.60 | 0.48 | 0.99 | 0.14 | -- | 0.30 | NO |
| **72607106** | 4:72607106 | C/T | 3’ UTR | 0.004 | 0 | 0 | 0 | -- | 0.08 | 0.19 | -- | 0.08 | 0.21 | NO |
| **rs2070742** | 4:72607161 | G/A | 3’ UTR | 0.007 | 0 | 0 | 0 | 0.60 | 0.32 | 0.13 | -- | -- | -- | NO |
| **72607217** | 4:72607217 | T/A | 3’ UTR | 0.004 | 0.050 | 0 | 0 | -- | 0.20 | 0.28 | -- | 0.22 | 0.30 | NO |
| **rs144499180** | 4:72607252 | G/C | 3’ UTR | 0.004 | 0 | 0 | 0 | 0.37 | 0.48 | 0.28 | -- | -- | -- | NO |
| **rs9016** | 4:72618296 | T/C | His445Arg | 0.008 | 0 | 0 | 0 | -- | 0.20 | 0.28 | -- | 0.22 | 0.30 | NO |
| **rs4588** | 4:72618323 | G/T | Thr436Lys | 0.271 | 0.400 | 0.300 | 0.200 | 0.49 | 0.32 | 0.22 | 0.08 | 0.06 | 0.03 | YES |
| **rs7041** | 4:72618334 | A/C | Asp432Glu | 0.424 | 0.300 | 0.550 | 0.500 | 0.004 | 0.0001 | 0.00 | 0.0001 | 0.0001 | 0.0001 | YES |
| **rs76803094** | 4:72620788 | C/T | Pro357 | 0.024 | 0.050 | 0.050 | 0 | 0.37 | 0.26 | 0.58 | 0.10 | 0.71 | 0.61 | NO |
| **rs780473721** | 4:72622531 | C/A | Cys311Phe | 0.007 | 0 | 0 | 0 | 0.20 | 0.32 | 0.13 | -- | -- | -- | NO |
| **rs4752** | 4:72622566 | A/G | Cys299 | 0.015 | 0.050 | 0 | 0 | 0.37 | 0.28 | 0.60 | 0.35 | 0.50 | 0.79 | NO |
| **rs41265647** | 4:72622601 | T/C | Asn288Asp | 0.004 | 0 | 0 | 0 | 0.37 | 0.27 | 0.19 | -- | -- | -- | NO |
| **rs114932951** | 4:72631184 | C/T | Glu146 | 0.012 | 0 | 0 | 0 | 0.13 | -- | 0.19 | 0.95 | 0.29 | 0.65 | NO |
| **rs769030498** | 4:72634150 | C/A | Leu43 | 0.008 | 0 | 0 | 0 | 0.13 | -- | 0.19 | 0.14 | -- | 0.21 | NO |
| ***IL13*** |  |  |  |  |  |  |  |  |  |  |  |  |  |  |
| **rs20541** | 5:131995964 | A/G | Gln144Arg | 0.197 | 0.100 | 0.150 | 0.100 | 0.29 | 0.48 | 0.82 | 0.06 | 0.81 | 0.40 | YES |
| **rs1295685** | 5:131996445 | A/G | 3`UTR | 0.200 | 0.100 | 0.150 | 0.100 | 0.29 | 0.45 | 0.95 | 0.06 | 0.54 | 0.35 | YES |
| **rs848** | 5:131996500 | A/C | 3´UTR | 0.216 | 0.150 | 0.150 | 0.100 | 0.27 | 0.29 | 0.89 | 0.03 | 0.36 | 0.21 | YES |
| **rs2069750** | 5:131996597 | G/C | 3´UTR | 0.004 | 0.050 | 0 | 0 | -- | 0.08 | 0.19 | -- | 0.08 | 0.21 | NO |
| **rs847** | 5:131996669 | T/C | 3´UTR | 0.204 | 0.100 | 0.150 | 0.100 | 0.19 | 0.50 | 0.98 | 0.02 | 0.45 | 0.13 | NO |
| **rs143032763** | 5:131996752 | T/G | 3´UTR | 0.019 | 0.100 | 0 | 0.100 | 0.21 | 0.19 | 0.09 | 0.10 | 0.07 | 0.03 | NO |
| ***IL4*** |  |  |  |  |  |  |  |  |  |  |  |  |  |  |
| **rs2070874** | 5:132009710 | C/T | 5´UTR | 0.139 | 0.150 | 0.050 | 0.200 | 0.16 | 0.26 | 0.31 | 0.01 | 0.54 | 0.07 | YES |
| **rs2243251** | 5:132009787 | A/G | Leu 15 | 0.004 | 0 | 0 | 0 | -- | -- | -- | 0.35 | 0.24 | 0.17 | NO |
| **rs35648164** | 5:132015555 | C/T | Asp 111 | 0.015 | 0.050 | 0 | 0.050 | 0.03 | 0.2 | 0.04 | 0.25 | 0.67 | 0.34 | NO |
| **rs565969000** | 5:132018487 | C/T | 3’ UTR | 0.004 | 0 | 0 | 0.050 | -- | -- | -- | 0.35 | 0.46 | 0.26 | NO |
| **rs71645921** | 5:132018876 | A/G | 3’ UTR | 0.004 | 0.050 | 0 | 0 | -- | 0.08 | 0.19 | -- | 0.08 | 0.21 | NO |
| **rs2243291** | 5:132018983 | G/C | 3’ UTR | 0.147 | 0.250 | 0.050 | 0.200 | 0.16 | 0.13 | 0.28 | 0.005 | 0.57 | 0.07 | NO |
| **rs2243292** | 5:132019221 | T/C | 3’ UTR | 0.004 | 0.050 | 0 | 0 | -- | -- | -- | 0.35 | 0.24 | 0.17 | NO |
| **rs559839441** | 5:132019246 | G/T | 3’ UTR | 0.007 | 0 | 0 | 0 | 0.37 | 0.48 | 0.28 | -- | -- | -- | NO |
| ***RXRB*** |  |  |  |  |  |  |  |  |  |  |  |  |  |  |
| **rs561418438** | 6:33161538 | G/A | 3´UTR | 0.004 | 0 | 0 | 0 | 0.37 | 0.48 | 0.28 | -- | -- | -- | NO |
| **rs770612532** | 6:33161590 | G/C | 3´UTR | 0.004 | 0 | 0 | 0 | 0.13 | -- | 0.19 | 0.14 | -- | 0.21 | NO |
| **33161660** | 6:33161660 | T/TG | 3´UTR | 0.079 | 0.200 | 0.150 | 0 | 0.07 | 0.04 | 0.04 | 0.66 | 0.03 | 0.14 | NO |
| **rs372089016** | 6:33161661 | A/G | 3´UTR | 0.231 | 0.250 | 0.150 | 0.100 | 0.03 | 0.004 | 0.001 | 0.35 | 0.20 | 0.29 | NO |
| **rs55678527** | 6:33161690 | A/T | 3´UTR | 0.019 | 0 | 0 | 0 | 0.37 | 0.43 | 0.71 | 0.19 | 0.73 | 0.80 | NO |
| **rs2072915** | 6:33162082 | T/A | 3´UTR | 0.321 | 0.250 | 0.350 | 0.350 | 0.40 | 0.02 | 0.06 | 0.71 | 0.07 | 0.22 | NO |
| **rs2744537** | 6:33162215 | A/C | 3´UTR | 0.230 | 0.250 | 0.150 | 0.150 | 0.08 | 0.003 | 0.003 | 0.72 | 0.18 | 0.51 | NO |
| **rs5030979** | 6:33162228 | A/T | 3´UTR | 0.096 | 0.150 | 0.050 | 0.200 | 1 | 0.67 | 0.83 | 0.27 | 0.77 | 0.33 | NO |
| **33162387** | 6:33162387 | T/C | 3´UTR | 0.004 | 0 | 0 | 0 | -- | 0.20 | 0.28 | -- | 0.22 | 0.30 | NO |
| **rs553683366** | 6:33162451 | G/T | 3´UTR | 0.008 | 0 | 0 | 0 | 0.13 | -- | 0.19 | 0.14 | -- | 0.21 | NO |
| **rs61669264** | 6:33162540 | C/G | Leu507 | 0.027 | 0.050 | 0 | 0.050 | 0.60 | 0.89 | 0.60 | 0.44 | 0.48 | 0.38 | NO |
| **rs6531** | 6:33163451 | G/A | Phe384 | 0.234 | 0.250 | 0.150 | 0.150 | 0.14 | 0.003 | 0.005 | 0.53 | 0.18 | 0.51 | NO |
| **33166184** | 6:33166184 | G/C | Asn180Lys | 0.004 | 0 | 0 | 0 | 0.37 | 0.48 | 0.28 | -- | -- | -- | NO |
| **rs772795938** | 6:33167066 | T/G | Pro 121 | 0.272 | 0.500 | 0.100 | 0.050 | 0.0001 | 0.002 | 0.0001 | 0.0001 | 0.01 | 0.0001 | NO |
| ***RXRA*** |  |  |  |  |  |  |  |  |  |  |  |  |  |  |
| **rs748087594** | 9:137293621 | A/G | Ile58Val | 0.008 | 0 | 0 | 0 | 0.13 | -- | 0.19 | 0.14 | -- | 0.21 | NO |
| **rs754761323** | 9:137300061 | G/A | Val116Ile | 0.004 | 0 | 0 | 0 | -- | -- | -- | 0.35 | 0.24 | 0.17 | NO |
| **rs137871665** | 9:137321000 | C/T | Ala319 | 0.012 | 0 | 0 | 0.050 | -- | 0.08 | 0.19 | 0.35 | 0.11 | 0.18 | NO |
| **rs1805348** | 9:137328442 | G/A | Ala457 | 0.026 | 0 | 0.050 | 0 | 0.07 | 0.41 | 0.14 | 0.19 | 0.80 | 0.44 | NO |
| **rs61751480** | 9:137328466 | C/T | 3´UTR | 0.008 | 0 | 0 | 0.050 | -- | -- | -- | 0.19 | 0.17 | 0 | NO |
| **rs376128806** | 9:137328502 | C/T | 3´UTR | 0.004 | 0.050 | 0 | 0 | -- | -- | -- | 0.35 | 0.46 | 0.26 | NO |
| **137328618** | 9:137328618 | A/C | 3´UTR | 0.008 | 0 | 0 | 0 | 0.13 | -- | 0.19 | 0.14 | -- | 0.21 | NO |
| **137328647** | 9:137328647 | G/C | 3´UTR | 0.008 | 0 | 0 | 0 | 0.13 | -- | 0.19 | 0.14 | -- | 0.21 | NO |
| **rs1805342** | 9:137328700 | C/T | 3´UTR | 0.004 | 0 | 0 | 0 | -- | -- | -- | 0.35 | 0.24 | 0.17 | NO |
| **137328845** | 9:137328845 | C/CGATGCT | 3´UTR | 0.004 | 0 | 0 | 0 | 0.37 | 0.48 | 0.28 | -- | -- | -- | NO |
| ***CYP2R1*** |  |  |  |  |  |  |  |  |  |  |  |  |  |  |
| **rs373226890** | 11:14899835 | G/T | His447Asp | 0.008 | 0.100 | 0 | 0 | -- | 0.08 | 0.19 | -- | 0.08 | 0.21 | NO |
| **rs140424660** | 11:14900714 | G/A | Leu426 | 0.004 | 0 | 0 | 0 | -- | 0.08 | 0.19 | -- | 0.08 | 0.21 | NO |
| **rs117913124** | 11:14900931 | G/A | Asp353 | 0.015 | 0.100 | 0 | 0.050 | 0.60 | 0.89 | 0.60 | 0.66 | 0.96 | 0.67 | YES |
| **rs12794714** | 11:14913575 | G/A | Ser 59 | 0.474 | 0.5 | 0.300 | 0.350 | 0.74 | 0.76 | 0.86 | 0.73 | -- | 0.88 | YES |
| ***MS4A2*** |  |  |  |  |  |  |  |  |  |  |  |  |  |  |
| **rs140124027** | 11:59856240 | T/G | Met1Arg | 0.004 | 0 | 0 | 0 | 0.37 | 0.48 | 0.28 | -- | -- | -- | NO |
| **rs35033981** | 11:59860922 | C/T | Thr143Met | 0.004 | 0.050 | 0 | 0.050 | 0.37 | 0.27 | 0.19 | -- | -- | -- | NO |
| **rs535630** | 11:59861532 | C/A | Asn211Lys | 0.008 | 0 | 0 | 0.050 | 0.12 | -- | 0.28 | 0.66 | 0.46 | 0.94 | NO |
| **rs569108** | 11:59863104 | A/G | Glu237Gly | 0.034 | 0 | 0.050 | 0.100 | 0.95 | 0.04 | 0.33 | 0.80 | 0.07 | 0.51 | YES |
| **rs512555** | 11:59863253 | C/T | 3´UTR | 0.034 | 0 | 0.050 | 0.100 | 0.95 | 0.04 | 0.33 | 0.80 | 0.07 | 0.51 | YES |
| **59863494** | 11:59863494 | AGGGTT/A | 3’ UTR | 0.008 | 0 | 0 | 0.100 | -- | -- | -- | 0.35 | 0.24 | 0.17 | NO |
| ***VDR*** |  |  |  |  |  |  |  |  |  |  |  |  |  |  |
| **rs11574139** | 12:48235555 | T/A | 3´UTR | 0.083 | 0.050 | 0.100 | 0 | 0.89 | 0.88 | 0.89 | 0.52 | 0.49 | 0.41 | NO |
| **rs760268626** | 12:48235556 | C/T | 3´UTR | 0.004 | 0 | 0 | 0 | -- | -- | -- | 0.35 | 0.46 | 0.26 | NO |
| **rs2853563** | 12:48235738 | C/T | 3´UTR | 0.091 | 0.050 | 0.100 | 0 | 0.73 | 0.77 | 0.71 | 0.71 | 0.62 | 0.59 | NO |
| **rs2853562** | 12:48236386 | T/A | 3´UTR | 0.478 | 0.450 | 0.188 | 0.611 | 0.14 | 0.44 | 0.16 | 0.26 | 0.22 | 0.25 | NO |
| **rs2544043** | 12:48236550 | G/C | 3´UTR | 0.121 | 0.150 | 0.250 | 0.125 | 0.98 | 0.84 | 0.96 | 0.36 | 0.43 | 0.26 | NO |
| **48236602** | 12:48236602 | C/T | 3´UTR | 0.004 | 0 | 0.0500 | 0 | 0.37 | 0.27 | 0.19 | -- | -- | -- | NO |
| **rs9729** | 12:48236623 | G/T | 3´UTR | 0.476 | 0.450 | 0.250 | 0.600 | 0.15 | 0.62 | 0.24 | 0.49 | 0.27 | 0.48 | NO |
| **rs11540149** | 12:48236664 | C/T | 3´UTR | 0.016 | 0.100 | 0 | 0 | 0.37 | 0.61 | 0.99 | 0.19 | 0.8 | 0.44 | NO |
| **rs11574134** | 12:48236863 | T/G | 3´UTR | 0.007 | 0 | 0.100 | 0 | 0.20 | 0.32 | 0.13 | -- | -- | -- | NO |
| **rs56382517** | 12:48237109 | A/G | 3´UTR | 0.019 | 0 | 0 | 0.050 | 0.21 | 0.19 | 0.09 | 0.10 | 0.12 | 0.04 | NO |
| **rs7954412** | 12:48237287 | T/C | 3´UTR | 0.004 | 0 | 0 | 0 | 0.37 | 0.48 | 0.28 | -- | -- | -- | NO |
| **rs11574129** | 12:48237303 | A/G | 3´UTR | 0.004 | 0 | 0 | 0 | 0.13 | -- | 0.19 | 0.14 | -- | 0.21 | NO |
| **rs556844112** | 12:48237652 | G/C | 3´UTR | 0.004 | 0 | 0 | 0 | -- | -- | -- | 0.35 | 0.46 | 0.26 | NO |
| **rs368484698** | 12:48237665 | C/T | 3´UTR | 0.008 | 0 | 0 | 0 | -- | 0.20 | 0.28 | -- | 0.22 | -- | NO |
| **48237732** | 12:48237732 | TGGCAG/T | 3´UTR | 0.008 | 0 | 0 | 0 | -- | 0.20 | 0.28 | -- | 0.22 | 0.30 | NO |
| **48237734** | 12:48237734 | GCA/G | 3´UTR | 0.028 | 0 | 0.050 | 0 | 0.21 | 0.19 | 0.09 | 0.26 | 0.43 | 0.15 | NO |
| **rs78783628** | 12:48237735 | CA/C | 3´UTR | 0.416 | 0.450 | 0.200 | 0.600 | 0.10 | 0.62 | 0.23 | 0.55 | 0.29 | 0.75 | NO |
| **rs11574125** | 12:48237736 | A/G | 3´UTR | 0.004 | 0 | 0 | 0 | -- | 0.20 | 0.28 | -- | 0.22 | 0.30 | NO |
| **rs527740586** | 12:48237743 | G/A | 3´UTR | 0.004 | 0 | 0 | 0 | -- | -- | -- | 0.35 | 0.46 | 0.26 | NO |
| **rs11574121** | 12:48237928 | G/A | 3´UTR | 0.004 | 0 | 0 | 0 | -- | 0.20 | 0.28 | -- | 0.22 | 0.30 | NO |
| **rs3858733** | 12:48237967 | T/G | 3´UTR | 0.083 | 0.050 | 0.100 | 0 | 0.89 | 0.88 | 0.89 | 0.52 | 0.49 | 0.41 | NO |
| **rs3847987** | 12:48238068 | C/A | 3´UTR | 0.107 | 0.150 | 0.100 | 0.050 | 0.08 | 0.72 | 0.19 | 0.12 | 0.76 | 0.25 | YES |
| **rs11574119** | 12:48238092 | G/C | 3´UTR | 0.094 | 0.150 | 0.150 | 0.050 | 0.59 | 0.95 | 0.72 | 0.52 | 0.22 | 0.26 | NO |
| **rs112308011** | 12:48238121 | A/G | 3´UTR | 0.004 | 0 | 0 | 0 | 0.37 | 0.48 | 0.28 | -- | -- | -- | NO |
| **rs739837** | 12:48238221 | G/T | 3’ UTR | 0.487 | 0.450 | 0.250 | 0.600 | 0.05 | 0.62 | 0.15 | 0.68 | 0.27 | 0.97 | YES |
| **rs731236** | 12:48238757 | A/G | Ile352 | 0.361 | 0.300 | 0.150 | 0.550 | 0.77 | 0.64 | 0.99 | 0.19 | 0.15 | 0.10 | YES |
| **48240506** | 12:48240506 | T/C | Met281Val | 0.004 | 0 | 0 | 0 | 0.13 | -- | 0.19 | 0.14 | -- | 0.21 | NO |
| **48251373** | 12:48251373 | C/T | Glu126Lys | 0.004 | 0 | 0 | 0 | -- | -- | -- | 0.35 | 0.46 | 0.26 | NO |
| **rs2228572** | 12:48272840 | G/A | Asp19 | 0.015 | 0 | 0 | 0 | 0.20 | 0.31 | 0.88 | -- | 0.03 | 0.10 | NO |
| **rs2228570** | 12:48272895 | A/G | Thr1Met | 0.349 | 0.250 | 0.250 | 0.400 | 0.99 | 0.40 | 0.64 | 0.47 | 0.85 | 0.83 | YES |
| ***CYP27B1*** |  |  |  |  |  |  |  |  |  |  |  |  |  |  |
| **rs8176351** | 12:58156510 | C/T | 3´UTR | 0.004 | 0 | 0 | 0 | 0.12 | -- | 0.28 | 0.14 | -- | 0.3 | NO |
| **rs8176350** | 12:58156848 | C/T | 3´UTR | 0.004 | 0 | 0 | 0 | 0.37 | 0.27 | 0.19 | -- | -- | -- | NO |
| **rs770746377** | 12:58158219 | A/G | Pro360Ser | 0.008 | 0 | 0 | 0 | 0.13 | -- | 0.19 | 0.14 | -- | 0.21 | NO |
| **rs8176345** | 12:58158558 | C/T | Leu314 | 0.019 | 0 | 0 | 0 | 0.45 | 0.32 | 0.88 | 0.25 | 0.46 | 0.56 | NO |
| **rs8176344** | 12:58159173 | C/G | Leu166Val | 0.004 | 0 | 0 | 0 | 0.37 | 0.27 | 0.19 | -- | -- | -- | YES |
| **rs771215877** | 12:58159866 | G/A | Arg104Trp | 0.004 | 0 | 0 | 0 | -- | -- | -- | 0.35 | 0.24 | 0.17 | NO |
| ***IL4R*** |  |  |  |  |  |  |  |  |  |  |  |  |  |  |
| **rs17548704** | 16:27353479 | C/T | Ser 36 | 0.030 | 0.050 | 0.100 | 0.050 | 0.71 | 0.90 | 0.90 | 0.52 | 0.95 | 0.68 | NO |
| **rs201852059** | 16:27353529 | A/G | Asn53Ser | 0.004 | 0 | 0.050 | 0 | 0.37 | 0.27 | 0.19 | -- | -- | -- | NO |
| **rs1805010** | 16:27356203 | A/G | Ile75Val | 0.468 | 0.550 | 0.400 | 0.500 | 0.84 | 0.22 | 0.64 | 0.60 | 0.30 | 0.45 | YES |
| **rs112497527** | 16:27356271 | T/C | Asp97 | 0.004 | 0 | 0 | 0 | 0.37 | 0.27 | 0.19 | -- | -- | -- | NO |
| **rs3024560** | 16:27356667 | T/G | 3`UTR | 0.407 | 0.550 | 0.250 | 0.350 | 0.39 | 0.03 | 0.11 | 0.80 | 0.24 | 0.48 | NO |
| **rs2234895** | 16:27357927 | C/T | Asn167 | 0.058 | 0 | 0.100 | 0.150 | 0.33 | 0.23 | 0.14 | 0.28 | 0.49 | 0.27 | NO |
| **rs775681273** | 16:27363876 | G/A | Val177Met | 0.004 | 0 | 0 | 0 | -- | 0.20 | 0.28 | -- | 0.22 | 0.30 | NO |
| **rs3024638** | 16:27367226 | C/G | Thr256 | 0.004 | 0 | 0.050 | 0 | 0.37 | 0.27 | 0.19 | -- | -- | -- | NO |
| **rs2234896** | 16:27370288 | C/G | Leu274 | 0.004 | 0.050 | 0 | 0 | -- | -- | -- | 0.35 | 0.24 | 0.17 | NO |
| **rs2234897** | 16:27373612 | T/C | Phe313 | 0.023 | 0 | 0 | 0 | 0.60 | 0.28 | 0.29 | 0.95 | 0.54 | 0.68 | NO |
| **rs6413500** | 16:27373833 | C/T | Ser387Leu | 0.015 | 0 | 0.050 | 0.050 | 0.97 | 0.19 | 0.61 | 0.66 | 0.46 | 0.90 | NO |
| **rs1805011** | 16:27373872 | A/C | Glu400Ala | 0.126 | 0.050 | 0.150 | 0.050 | 0.65 | 0.54 | 0.42 | 0.37 | 0.48 | 0.23 | YES |
| **rs2234898** | 16:27373915 | G/T | Leu414 | 0.126 | 0.050 | 0.150 | 0.050 | 0.65 | 0.54 | 0.42 | 0.37 | 0.48 | 0.23 | NO |
| **rs1805012** | 16:27373964 | T/C | Cys431Arg | 0.115 | 0.050 | 0.150 | 0.050 | 0.66 | 0.51 | 0.41 | 0.39 | 0.52 | 0.26 | YES |
| **rs2234899** | 16:27373966 | C/T | Cys431 | 0.008 | 0 | 0 | 0 | -- | 0.20 | 0.28 | 0.35 | 0.67 | 0.94 | NO |
| **rs2234900** | 16:27373972 | T/C | Leu433 | 0.134 | 0.050 | 0.150 | 0.050 | 0.65 | 0.51 | 0.40 | 0.40 | 0.44 | 0.24 | NO |
| **rs1805013** | 16:27373980 | C/T | Ser436Leu | 0.027 | 0 | 0.050 | 0 | 0.45 | 0.88 | 0.51 | 0.52 | 0.95 | 0.59 | YES |
| **rs1805015** | 16:27374180 | T/C | Ser503Pro | 0.153 | 0.050 | 0.200 | 0.050 | 0.67 | 0.53 | 0.43 | 0.39 | 0.49 | 0.24 | YES |
| **27374260** | 16:27374260 | G/A | Glu529 | 0.004 | 0 | 0 | 0 | 0.37 | 0.27 | 0.19 | -- | -- | -- | NO |
| **rs1801275** | 16:27374400 | A/G | Gln551Arg | 0.202 | 0.050 | 0.200 | 0.050 | 0.86 | 0.53 | 0.99 | 0.98 | 0.36 | 0.69 | YES |
| **rs3024677** | 16:27374408 | G/A | Val579Ile | 0.004 | 0 | 0 | 0 | -- | -- | -- | 0.35 | 0.46 | 0.26 | NO |
| **rs3024678** | 16:27374696 | C/T | Pro679Ser | 0.011 | 0 | 0.050 | 0 | 0.21 | 0.27 | 0.49 | 0.04 | -- | 0.07 | NO |
| **rs1805016** | 16:27374927 | T/G | Ser752Ala | 0.046 | 0 | 0.050 | 0 | 0.34 | 0.49 | 0.30 | 0.43 | 0.44 | 0.34 | NO |
| **rs1805014** | 16:27375029 | T/C | Ser786Pro | 0.004 | 0 | 0 | 0 | -- | -- | -- | 0.35 | 0.46 | 0.26 | NO |
| **rs2074570** | 16:27375157 | T/C | 3´UTR | 0.038 | 0 | 0 | 0 | 0.71 | 0.36 | 0.44 | 0.52 | 0.29 | 0.31 | NO |
| **rs3024680** | 16:27375159 | T/C | 3´UTR | 0.012 | 0 | 0 | 0 | -- | 0.20 | 0.28 | 0.18 | 0.95 | 0.54 | NO |
| **rs2234924** | 16:27375239 | C/G | 3´UTR | 0.027 | 0 | 0.050 | 0 | 0.45 | 0.88 | 0.51 | 0.52 | 0.95 | 0.59 | NO |
| **rs188457282** | 16:27375354 | G/A | 3´UTR | 0.004 | 0 | 0.050 | 0 | 0.37 | 0.27 | 0.19 | -- | -- | -- | NO |
| **rs77218469** | 16:27375450 | G/C | 3´UTR | 0.004 | 0 | 0 | 0 | -- | -- | -- | 0.35 | 0.46 | 0.26 | NO |
| **rs1049631** | 16:27375542 | G/A | 3´UTR | 0.465 | 0.600 | 0.450 | 0.450 | 0.0007 | 0.62 | 0.02 | 0.21 | 0.24 | 0.91 | NO |
| **rs8832** | 16:27375787 | A/G | 3´UTR | 0.457 | 0.600 | 0.400 | 0.450 | 0.0002 | 0.60 | 0.004 | 0.23 | 0.24 | 0.94 | YES |
| **rs8674** | 16:27375889 | C/T | 3´UTR | 0.034 | 0 | 0.050 | 0 | 0.19 | 0.46 | 0.20 | 0.23 | 0.54 | 0.26 | NO |
| **rs1029489** | 16:27376217 | A/G | 3’ UTR | 0.377 | 0.550 | 0.300 | 0.450 | 0.001 | 0.24 | 0.007 | 0.29 | 0.09 | 0.63 | YES |
| **rs3024697** | 16:27376483 | G/C | 3’ UTR | 0.004 | 0 | 0 | 0 | 0.12 | -- | 0.28 | 0.14 | -- | 0.30 | NO |
| ***CYP24A1*** |  |  |  |  |  |  |  |  |  |  |  |  |  |  |
| **rs4811494** | 20:52769984 | C/T | 3’ UTR | 0.227 | 0.200 | 0.150 | 0.2000 | 0.58 | 0.08 | 0.54 | 0.46 | 0.56 | 0.87 | NO |
| **52770054** | 20:52770054 | C/CAT | 3´UTR | 0.308 | 0.300 | 0.300 | 0.100 | 0.99 | 0.23 | 0.74 | 0.84 | 0.16 | 0.44 | NO |
| **rs11907350** | 20:52770439 | G/A | 3´UTR | 0.038 | 0.050 | 0 | 0 | 0.17 | 0.84 | 0.32 | 0.43 | 0.74 | 0.55 | NO |
| **rs6022987** | 20:52770596 | G/C | 3´UTR | 0.317 | 0.300 | 0.400 | 0.350 | 0.28 | 0.42 | 0.29 | 0.98 | 0.14 | 0.50 | NO |
| **rs4809957** | 20:52771171 | A/G | 3´UTR | 0.231 | 0.200 | 0.150 | 0.200 | 0.58 | 0.08 | 0.54 | 0.71 | 0.26 | 0.98 | NO |
| **rs2762934** | 20:52771261 | G/A | 3´UTR | 0.199 | 0.200 | 0.250 | 0.150 | 0.81 | 0.32 | 0.42 | 0.81 | 0.16 | 0.36 | YES |
| **rs114368325** | 20:52774675 | G/A | Trp396Arg | 0.004 | 0 | 0 | 0 | 0.13 | -- | 0.19 | 0.14 | -- | 0.21 | NO |
| **rs2296239** | 20:52775528 | C/T | Pro 375 | 0.233 | 0.200 | 0.150 | 0.200 | 0.48 | 0.07 | 0.39 | 0.71 | 0.29 | 1 | NO |
| **52775646** | 20:52775646 | ATTAGACTG/A | Exón 8 | 0.004 | 0 | 0 | 0.050 | -- | -- | -- | 0.35 | 0.46 | 0.26 | NO |
| **rs6068816** | 20:52781091 | C/T | Thr248 | 0.107 | 0.050 | 0.050 | 0.150 | 0.66 | 0.62 | 0.50 | 0.89 | 0.42 | 0.67 | NO |
| **rs2296241** | 20:52786219 | G/A | Ala 184 | 0.455 | 0.500 | 0.550 | 0.450 | 0.92 | 0.23 | 0.39 | 0.97 | 0.50 | 0.83 | YES |
| **rs35873579** | 20:52788190 | G/A | Arg157Trp | 0.027 | 0.050 | 0 | 0 | 0.98 | 0.31 | 0.51 | 0.95 | 0.54 | 0.68 | NO |
| **52789466** | 20:52789466 | CCTT/C | Exón 2 | 0.007 | 0 | 0 | 0 | 0.37 | 0.61 | 0.99 | -- | 0.22 | 0.30 | NO |
| **rs61755338** | 20:52789885 | A/C | Gly 78 | 0.015 | 0.050 | 0 | 0 | 0.36 | 0.16 | 0.51 | 0.35 | 0.32 | 0.67 | NO |
| **rs61749689** | 20:52790005 | C/A | Pro 38 | 0.019 | 0.050 | 0 | 0 | 0.20 | 0.31 | 0.88 | 0.35 | 0.32 | 0.67 | NO |
| ***IL13RA1*** |  |  |  |  |  |  |  |  |  |  |  |  |  |  |
| **rs555458472** | X:117861629 | C/T | Syn Leu10 | 0.008 | 0 | 0 | 0 | -- | -- | -- | 0.35 | 0.24 | 0.17 | NO |
| **rs144170222** | X:117907882 | C/T | Leu350 | 0.023 | 0.050 | 0 | 0 | 0.60 | 0.48 | 0.99 | 0.14 | 0.29 | 0.17 | NO |
| **rs2495636** | X:117925898 | A/G | 3´UTR | 0.172 | 0.100 | 0.250 | 0.300 | 0.79 | 0.26 | 0.43 | 0.87 | 0.26 | 0.49 | YES |
| **rs141664428** | X:117926106 | A/G | 3´UTR | 0.007 | 0 | 0 | 0 | 0.37 | 0.27 | 0.19 | -- | -- | -- | NO |
| **rs2254758** | X:117926379 | C/T | 3´UTR | 0.172 | 0.100 | 0.250 | 0.300 | 0.79 | 0.26 | 0.43 | 0.87 | 0.26 | 0.49 | NO |
| **117926720** | X:117926720 | C/CATG | 3´UTR | 0.130 | 0.100 | 0.200 | 0.250 | 0.78 | 0.30 | 0.45 | 0.90 | 0.35 | 0.60 | NO |
| **rs2254672** | X:117926781 | T/G | 3´UTR | 0.172 | 0.100 | 0.250 | 0.300 | 0.79 | 0.26 | 0.43 | 0.87 | 0.26 | 0.49 | YES |
| **117926915** | X:117926915 | C/T | 3´UTR | 0.004 | 0 | 0 | 0 | -- | -- | -- | 0.35 | 0.46 | 0.26 | NO |
| **rs767508589** | X:117926946 | G/A | 3´UTR | 0.008 | 0 | 0 | 0 | 0.37 | 0.48 | 0.28 | 0.35 | 0.46 | 0.26 | NO |
| **rs141481173** | X:117927441 | A/T | 3´UTR | 0.007 | 0 | 0 | 0 | 0.37 | 0.27 | 0.19 | -- | -- | -- | NO |
| **rs145266690** | X:117927495 | C/T | 3´UTR | 0.008 | 0 | 0 | 0 | -- | -- | -- | 0.35 | 0.24 | 0.17 | NO |

Table S4. SNVs significantly associated with SNIUAA patients manifesting cutaneous symptoms (adjusted by sex).(C: Codominant model, D:Dominant model and R: Recessive model).

| **Model** | **Genotype** | **Other symptoms**  **(N, Freq.)** | **Cutaneous symptoms**  **(N, Freq.)** | **OR (95% CI)** | **P-value**  **Crude/Bonf** |
| --- | --- | --- | --- | --- | --- |
| ***CYP2R1*_rs12794714** |  |  |  |  |  |
| CD | G/G | 29 (0.204) | 52 (0.306) | 1 |  |
|  | G/A | 74 (0.521) | 80 (0.471) | 0.60 (0.35-1.05) | 0.072/0.144 |
|  | A/A | 39 (0.275) | 38 (0.224) | 0.54 (0.28-1.02) | 0.058/0.116 |
| D | G/G | 29 (0.204) | 52 (0.306) | 1 |  |
|  | G/A-A/A | 113 (0.796) | 118 (0.694) | 0.58 (0.34-0.98) | 0.039/0.078 |
| R | G/G-G/A | 103 (0.725) | 132 (0.777) | 1 |  |
|  | A/A | 39 (0.275) | 38 (0.224) | 0.76 (0.45-1.27) | 0.290/0.580 |
| ***CYP24A1*_rs2762934** |  |  |  |  |  |
| CD | G/G | 77 (0.55) | 111 (0.657) | 1 |  |
|  | G/A | 56 (0.40) | 56 (0.331) | 0.69 (0.43-1.11) | 0.124/0.248 |
|  | A/A | 7 (0.05) | 2 (0.012) | 0.19 (0.04-0.96) | 0.045/0.090 |
| D | G/G | 77 (0.55) | 111 (0.657) | 1 |  |
|  | G/A-A/A | 63 (0.45) | 58 (0.343) | 0.64 (0.40-1.01) | 0.053/0.106 |
| R | G/G-G/A | 133 (0.95) | 167 (0.988) | 1 |  |
|  | A/A | 7 (0.05) | 2 (0.012) | 0.22 (0.05-1.10) | 0.041/0.082 |

Other symptoms: anaphylaxis, rhinitis, asthma, exanthema, erythema, eczema, glottic edema, rash or pruritus. Bonf: Bonferroni correction.

Table S5. SNVs significantly associated with SNIUAA patients manifesting anaphylaxis. (adjusted by sex).(C: Codominant model, D:Dominant model and R: Recessive model).

| **Model** | **Genotype** | **Other symptoms**  **(N, Freq.)** | **Anaphylaxis**  **(N, Freq.)** | **OR (95% CI)** | **P-value**  **Crude/Bonf** |
| --- | --- | --- | --- | --- | --- |
| ***GC*_rs139523630** |  |  |  |  |  |
| CD | T/T | 195 (0.99) | 94 (0.931) | 1 |  |
|  | T/C | 2 (0.01) | 7 (0.069) | 7.27  (1.48-35.71) | **0.006/0.024** |
| ***IL4*_rs2243291** |  |  |  |  |  |
| CD | G/G | 129 (0.642) | 75 (0.735) | 1 |  |
|  | G/C | 61 (0.304) | 26 (0.255) | 0.73  (0.43-1.26) | 0.260/1.000 |
|  | C/C | 11 (0.055) | 1 (0.01) | 0.16  (0.02-1.30) | 0.088/0.352 |
| D | G/G | 129 (0.642) | 75 (0.735) | 1 |  |
|  | G/C-C/C | 72 (0.358) | 27 (0.265) | 0.65  (0.38-1.10) | 0.100/0.400 |
| R | G/G-G/C | 190 (0.945) | 101 (0.99) | 1 |  |
|  | C/C | 11 (0.055) | 1 (0.01) | 0.18  (0.02-1.42) | 0.042/0.168 |
| ***IL4R*_rs1805013** |  |  |  |  |  |
| CD | C/C | 191 (0.945) | 105 (0.991) | 1 |  |
|  | C/T | 10 (0.05) | 1 (0.009) | 0.18  (0.02-1.44) | 0.106/0.424 |
|  | T/T | 1 (0.005) | 0 (0) | 0.00  (0.00-NA) | -- |
| D | C/C | 191 (0.945) | 105 (0.991) | 1 |  |
|  | C/T-T/T | 11 (0.055) | 1 (0.009) | 0.17  (0.02-1.30) | 0.031/0.124 |
| R | C/C-C/T | 201 (0.995) | 106 (1) | 1 |  |
|  | T/T | 1 (0.005) | 0 (0) | 0.00  (0.00-NA) | 0.370/1.000 |
| ***CYP24A1*_rs2762934** |  |  |  |  |  |
| CD | G/G | 134 (0.66) | 54 (0.509) | 1 |  |
|  | G/A | 66 (0.325) | 46 (0.434) | 1.74  (1.06-2.85) | 0.027/0.108 |
|  | A/A | 3 (0.015) | 6 (0.057) | 5.12  (1.23-21.30) | 0.025/0.100 |

Other symptoms: urticaria, angioedema, rhinitis, asthma, exanthema, erythema, eczema, glottic edema, rash or pruritus. Bonf: Bonferroni correction.

Table S6. SNPs significantly associated with the risk of manifesting cutaneous symptoms in CR NSAIDs patients (adjusted by sex)..

(C: Codominant model, D:Dominant model and R: Recessive model).

| **Model** | **Genotype** | **Other symptoms**  **(N, Freq.)** | **Cutaneous symptoms**  **(N, Freq.)** | **OR (95% CI)** | **P-value**  **Crude/Bonf** |
| --- | --- | --- | --- | --- | --- |
| ***GC*_rs139523630** |  |  |  |  |  |
| CD | T/T | 127 (0.948) | 319 (0.985) | 1 |  |
|  | T/C | 6 (0.045) | 5 (0.015) | 0.32  (0.10-1.07) | 0.065/0.195 |
|  | C/C | 1 (0.008) | 0 (0) | 0.00 (0.00-NA) | -- |
| D | T/T | 127 (0.948) | 319 (0.985) | 1 |  |
|  | T/C-C/C | 7 (0.052) | 5 (0.015) | 0.28  (0.09-0.89) | 0.031/0.093 |
| R | T/T-T/C | 133 (0.992) | 324 (1.00) | 1 |  |
|  | C/C | 1 (0.008) | 0 (0) | 0.00 (0.00-NA) | 0.130/0.390 |
| ***IL4R*_rs1049631** |  |  |  |  |  |
| CD | A/A | 33 (0.241) | 111 (0.332) | 1 |  |
|  | A/G | 73 (0.533) | 152 (0.455) | 0.62  (0.38-1.00) | 0.048/0.144 |
|  | G/G | 31 (0.226) | 71 (0.213) | 0.67  (0.38-1.19) | 0.170/0.510 |
| D | A/A | 33 (0.241) | 111 (0.332) | 1 |  |
|  | A/G-G/G | 104 (0.759) | 223 (0.668) | 0.63  (0.40-0.99) | 0.043/0.129 |
| R | A/A-A/G | 106 (0.774) | 263 (0.787) | 1 |  |
|  | G/G | 31 (0.226) | 71 (0.213) | 0.91  (0.56-1.47) | 0.700/1.000 |
| ***CYP24A1*_rs2762934** |  |  |  |  |  |
| CD | G/G | 50 (0.476) | 118 (0.599) | 1 |  |
|  | G/A | 47 (0.448) | 71 (0.360) | 0.62  (0.38-1.02) | 0.060/0.180 |
|  | A/A | 8 (0.076) | 8 (0.041) | 0.40  (0.14-1.13) | 0.083/0.249 |
| D | G/G | 50 (0.476) | 118 (0.599) | 1 |  |
|  | G/A-A/A | 55 (0.524) | 79 (0.401) | 0.59  (0.36-0.95) | 0.030/0.090 |
| R | G/G-G/A | 97 (0.924) | 189 (0.959) | 1 |  |
|  | A/A | 8 (0.076) | 8 (0.041) | 0.49  (0.18-1.35) | 0.170/0.510 |

Other symptoms: anaphylaxis, rhinitis, asthma, exanthema, erythema, eczema, glottic edema, rash or pruritus.

Cutaneous symptoms: Urticaria and/or angioedema.

Bonf: Bonferroni correction.

Table S7. SNVs significantly associated with the risk of manifesting anaphylaxis in CR NSAIDs patients anaphylaxis (adjusted by sex)..

(C: Codominant model, D:Dominant model and R: Recessive model).

| **Model** | **Genotype** | **Other symptoms**  **(N, Freq.)** | **Anaphylaxis**  **(N, Freq.)** | **OR (95% CI)** | **P-value** |
| --- | --- | --- | --- | --- | --- |
| ***FCER1G*_rs11421** |  |  |  |  |  |
| CD | T/T | 403 (0.682) | 30 (0.667) | 1 |  |
|  | T/G | 170 (0.288) | 10 (0.222) | 0.79 (0.38-1.65) | 0.529 |
|  | G/G | 18 (0.03) | 5 (0.111) | 3.74 (1.30-10.79) | **0.015** |
| D | T/T | 403 (0.682) | 30 (0.667) | 1 |  |
|  | T/G-G/G | 188 (0.318) | 15 (0.333) | 1.07 (0.56-2.04) | 0.832 |
| R | T/T-T/G | 573 (0.97) | 40 (0.889) | 1 |  |
|  | G/G | 18 (0.03) | 5 (0.111) | 3.99 (1.41-11.32) | **0.021** |

Other symptoms: urticaria, angioedema, rhinitis, asthma, exanthema, erythema, eczema, glottic edema, rash or pruritus.

Table S8. Differences in the SNVs associated with SNIUAA and CR NSAIDs patients (adjusted by sex). (C: Codominant model, D: Dominant model, and R: Recessive model).

| **HS vs HC** | | | | | |
| --- | --- | --- | --- | --- | --- |
| **Gene/SNP** | **Genotype** | **CR NSAIDs** | **SNIUAA** | **OR (95% CI)** | **p-value**  **(crude/BH)** |
|  |  | **(N, Freq.)** | **(N, Freq.)** |  |  |
| ***VDR*** |  |  |  |  |  |
| **rs78783628** |  |  |  |  |  |
| CD | -/- | 107 (0.296) | 71 (0.231) | 1.00 |  |
|  | A/- | 174 (0.482) | 148 (0.48) | 0.81 (0.56-1.18) | 0.127/0.797 |
|  | A/A | 80 (0.222) | 89 (0.289) | 0.61 (0.39-0.93) | 0.022/0.581 |
| D | -/- | 107 (0.296) | 71 (0.231) | 1.00 |  |
|  | A/--A/A | 254 (0.704) | 237 (0.77) | 0.73 (0.52-1.04) | 0.082/0.838 |
| R | -/--A/- | 281 (0.778) | 219 (0.711) | 1.00 |  |
|  | A/A | 80 (0.222) | 89 (0.289) | 0.69 (0.49-0.99) | 0.041/0.297 |
| **rs739837** |  |  |  |  |  |
| CD | T/T | 166 (0.285) | 70 (0.229) | 1.00 |  |
|  | T/G | 287 (0.492) | 147 (0.482) | 0.84 (0.59-1.18) | 0.306/0.923 |
|  | G/G | 130 (0.223) | 88 (0.289) | 0.63 (0.43-0.93) | 0.019/0.581 |
| D | T/T | 166 (0.285) | 70 (0.229) | 1.00 |  |
|  | T/G-G/G | 417 (0.715) | 235 (0.77) | 0.76 (0.55-1.05) | 0.089/0.838 |
| R | T/T-T/G | 453 (0.777) | 217 (0.712) | 1.00 |  |
|  | G/G | 130 (0.223) | 88 (0.289) | 0.71 (0.51-0.97) | 0.032/0.297 |
| **rs731236** |  |  |  |  |  |
| CD | A/A | 229 (0.38) | 123 (0.398) | 1.00 |  |
|  | A/G | 281 (0.466) | 156 (0.505) | 0.98 (0.73-1.32) | 0.913/0.973 |
|  | G/G | 93 (0.154) | 30 (0.097) | 1.65 (1.03-2.63) | 0.036/0.581 |
| D | A/A | 229 (0.38) | 123 (0.398) | 1.00 |  |
|  | A/G-G/G | 374 (0.62) | 186 (0.602) | 1.09 (0.82-1.45) | 0.538/0.845 |
| R | A/A-A/G | 510 (0.846) | 279 (0.903) | 1.00 |  |
|  | G/G | 93 (0.154) | 30 (0.097) | 1.67 (1.07-2.58) | 0.019/0.260 |
| ***IL4R*** |  |  |  |  |  |
| **rs1805013** |  |  |  |  |  |
| CD | C/C | 433 (0.921) | 298 (0.961) | 1.00 |  |
|  | C/T | 37 (0.079) | 11 (0.035) | 2.33 (1.17-4.65) | 0.016/0.581 |
|  | T/T | 0 (0) | 1 (0.003) | 0.00 (0.00-NA) | 1.000/1.000 |
| D | C/C | 433 (0.921) | 298 (0.961) | 1.00 |  |
|  | C/T-T/T | 37 (0.079) | 12 (0.039) | 2.14 (1.10-4.18) | 0.019/0.838 |
| R | C/C-C/T | 470 (1) | 309 (0.997) | 1.00 |  |
|  | T/T | 0 (0) | 1 (0.003) | 0.00 (0.00-NA) | 0.190/0.641 |

BH:Benjamini-Hochberg correction

Table S9. Association of vitamin D levels with demographic and clinical data of individuals included in the study.

| **Variables** | | **N, Mean± standard deviation (µ ± σ) (ng/ml), p- value (Kruskal-Wallis)** | | | | | | | | | | | |
| --- | --- | --- | --- | --- | --- | --- | --- | --- | --- | --- | --- | --- | --- |
|  |  | Non atopic controls | | | Atopic controls | | | SNIUAA | | | CR NSAIDs | | |
|  |  | N | (µ ± σ) | p- value | N | (µ ± σ) | p- value | N | (µ ± σ) | p- value | N | **(µ ± σ)** | **p- value** |
| Sex | M | 71 | 17.84±3.91 | 0.070 | 66 | 24.82±8.15 | 0.341 | 79 | 25.98±10.12 | 0.323 | 72 | 21.30±8.25 | 0.344 |
|  | F | 97 | 19.20±4.66 |  | 104 | 23.30±8.28 |  | 123 | 24.66±9.37 |  | 88 | 22.81±10.18 |  |
| History of atopy | No | 168 | 18.63±4.40 | - | 0 | - | - | 158 | 25.53±10.09 | 0.447 | 122 | 23.65±9.50 | **0.003** |
|  | Yes | 0 | - |  | 170 | 23.89±8.24 |  | 44 | 23.89±7.95 |  | 32 | 18.40±6.93 |  |
| History of urticaria | No | - | - | - | - | - | - | 200 | 25.22±9.71 | 0.618 | 97 | 26.19±8.92 | - |
|  | Yes | - | - |  | - | - |  | 2 | 21.07±1.80 |  | 0 | - |  |
| Anaphylaxis | No | - | - | - | - | - | - | 129 | 25.48±.75 | 0.503 | 133 | 22.35±9.19 | 0.241 |
|  | Yes | **-** | **-** |  | **-** | **-** |  | **72** | **24.70±9.61** |  | 25 | 21.28±10.47 |  |
| Cutaneous | No | - | - | **-** | - | - | - | 89 | 24.49±9.34 | 0.357 | 56 | 23.34±9.17 | 0.188 |
|  | Yes | - | - |  | - | - |  | 112 | 25.76±9.96 |  | 102 | 21.54±9.47 |  |
| Respiratory | No | - | - | - | - | - | - | 200 | 25.15±9.68 | 0.190 | 142 | 22.13±9.58 | 0.454 |
|  | Yes | - | - |  | - | - |  | 1 | 35.44 |  | 16 | 22.63±7.55 |  |
| Blended | No | - | - | - | - | - | - | 96 | 24.19±9.25 | 0.300 | 143 | 21.61±9.40 | **0.002** |
|  | Yes | - | - |  | - | - |  | 1 | 29.21 |  | 13 | 28.67±7.16 |  |
| Other symptoms | No | - | - | - | - | - | - | 100 | 24.34±9.16 | 0.791 | 143 | 21.61±9.40 | 0.959 |
|  | Yes | - | - |  | - | - |  | 2 | 22.49±9.20 |  | 2 | 20.21±4.47 |  |

**Table S10**. SNVs statistically significant associated with vitamin D levels (adjusted by sex).

|  | **All subjects** | | **Non atopic controls** | | **Atopic controls** | | **SNIUAA** | | **CR NSAIDs** | |
| --- | --- | --- | --- | --- | --- | --- | --- | --- | --- | --- |
| **Gene_SNP** | **Vitamin D N,(Mean±SD)** | **p-value (GLM)/Bonf** | **Vitamin D N,(Mean±SD)** | **p-value (GLM)/Bonf** | **Vitamin D N,(Mean±SD)** | **p-value (GLM)/Bonf** | **Vitamin D N,(Mean±SD)** | **p-value (GLM)/Bonf** | **Vitamin D N,(Mean±SD)** | **p-value (GLM)/Bonf** |
| ***FCER1G*** |  |  |  |  |  |  |  |  |  |  |
| **rs11421** |  |  |  |  |  |  |  |  |  |  |
| T/T | 1215,(22.50.±8.21.) |  | 209,(18.93.±4.73.) |  | 351,(22.32.±7.24.) |  | 218,(25.06.±9.04.) |  | 437,(22.39.±9.23.) |  |
| T/C | 499,(23.74±9.28) | 0.109/0.985 | 85,(18.82±4.56) | 0.717./1.000. | 147,(26.71±8.50) | **0.001./0..005** | 85,(25.37±10.86) | 0.796./1.000. | 182,(20.87±8.38) | 0.314./1.000. |
| C/C | 61,(24.16±13.34) | 0.346./1.000. | 12,(16.09±2.88) | 0.103./0.923. | 16,(28.90±18.25) | 0.110./0.987 | 10,(25.83±12.57) | 0.769./1.000. | 23,(28.31±18.17) | 0.144./1.000. |
| ***GC*** |  |  |  |  |  |  |  |  |  |  |
| **rs4588** |  |  |  |  |  |  |  |  |  |  |
| G/G | 590,(23.22±9.62.) |  | 138,(19.74.±4.33.) |  | 0,n.a. | n.a. | 143,(26.07±10.72.) |  | 309,(22.98.±10.79.) |  |
| G/T | 533,(21.36±7.95) | **0.022./0.198.** | 151,(17.46±4.29) | **0.002./0.014.** | 0,n.a. | n.a. | 133,(24.99±8.93) | 0.504./1.000. | 249,(21.22±7.80) | 0.262./1.000. |
| T/T | 111,(21.58±7.42) | 0.238./1.000. | 28,(18.69±3.11) | 0.490./1.000. | 0,n.a. | n.a. | 26,(21.70±8.30) | 0.114/1.000. | 57,(23.29±8.34) | 0.884./1.000. |
| **rs7041** |  |  |  |  |  |  |  |  |  |  |
| **C/C** | 347,(23.80.±10.28.) |  | 69,(20.65.±3.96) |  | 0,n.a. | n.a. | 92,(25.78.±11.53) |  | 186,(23.55±11.17) |  |
| C/A | 625,(21.75±8.04) | **0.024**/0.214 | 166,(18.14±4.36) | **0.003/0.028** | 0,n.a. | n.a. | 160,(25.14±8.51) | 0.720/1.000. | 299,(21.17±8.86) | 0.186/1.000. |
| A/A | 272,(21.61±8.12) | **0.049.**/0.444 | 79,(17.93±4.22) | **0.014./0.122** | 0,n.a. | n.a. | 62,(24.33±9.97) | 0.509/1.000. | 131,(22.09±7.71) | 0.451/1.000. |
| ***IL4R*** |  |  |  |  |  |  |  |  |  |  |
| **rs1805010** |  |  |  |  |  |  |  |  |  |  |
| A/A | 257,(22.74.±8.43.) |  | 85,(20.03±4.13.) |  | 0,n.a. |  | 96,(25.13.±9.65.) |  | 76,(21.16.±8.55.) |  |
| A/G | 377,(21.77±9.35) | 0.303./1.000. | 131,(17.24±4.24) | **0.0004/0.004** | 0,n.a. | n.a. | 128,(24.41±9.87) | 0.539./1.000. | 118,(23.07±10.73) | 0.353/1.000. |
| G/G | 149,(21.40±6.87) | 0.263/1.000. | 52,(19.11±4.22) | 0.309/1.000. | 0,n.a. | n.a. | 49,(23.25±7.87) | 0.394./1.000. | 48,(21.82±7.48) | 0.751./1.000. |
| **rs3024678** |  |  |  |  |  |  |  |  |  |  |
| C/C | 1576,(22.53.±8.44.) |  | 373,(18.43.±4.40.) |  | 433,(23.95±8.20.) |  | 301,(24.62.±8.96.) |  | 469,(22.20.±9.49.) |  |
| C/T | 53,(26.43±12.72) | **0.016./0.148.** | 16,(19.26±3.89) | 0.627./1.000. | 14,(24.94±8.91) | 0.815./1.000. | 12,(36.52±16.67) | **0.0002/0.002** | 11,(21.81±8.29) | 0.969/1.000. |
| T/T | 3,(20.34±2.37) | 0.647/1.000. | 2,(19.00±0.71) | 0.992./1.000. | 1,(23.02±0.00) | 0.959./1.000. | 0,n.a. | n.a. | 0,n.a. | n.a. |
| **rs1049631** |  |  |  |  |  |  |  |  |  |  |
| A/A | 498,(22.61.±7.52.) |  | 112,(19.55.±4.24.) |  | 138,(23.41.±7.74.) |  | 103,(23.72.±8.31.) |  | 145,(22.53.±7.61.) |  |
| A/G | 765,(22.96±9.37) | 0.645/1.000. | 183,(17.33±4.24) | **0.006./0.053.** | 203,(24.10±7.99) | 0.703./1.000. | 152,(26.06±10.38) | 0.140./1.000. | 227,(22.59±10.50) | 0.912./1.000. |
| G/G | 314,(22.58±9.21) | 0.982./1.000. | 73,(18.66±4.09) | 0.267./1.000. | 83,(24.66±9.40) | 0.560/1.000. | 54,(25.64±10.72) | 0.382/1.000. | 104,(21.20±9.65) | 0.631/1.000. |

GLM: Generalized linear model; Bonf: Bonferroni correction
