# Supplementary material for: Genetic and serum biomarkers of NSAID hypersensitivity reactions
Source: Front Pharmacol. 2025 Oct 2;16:1502755. doi: 10.3389/fphar.2025.1502755 (PMC12528179; doi:10.3389/fphar.2025.1502755)
Supplement: Supplementary file 2 [file Image1.pdf]

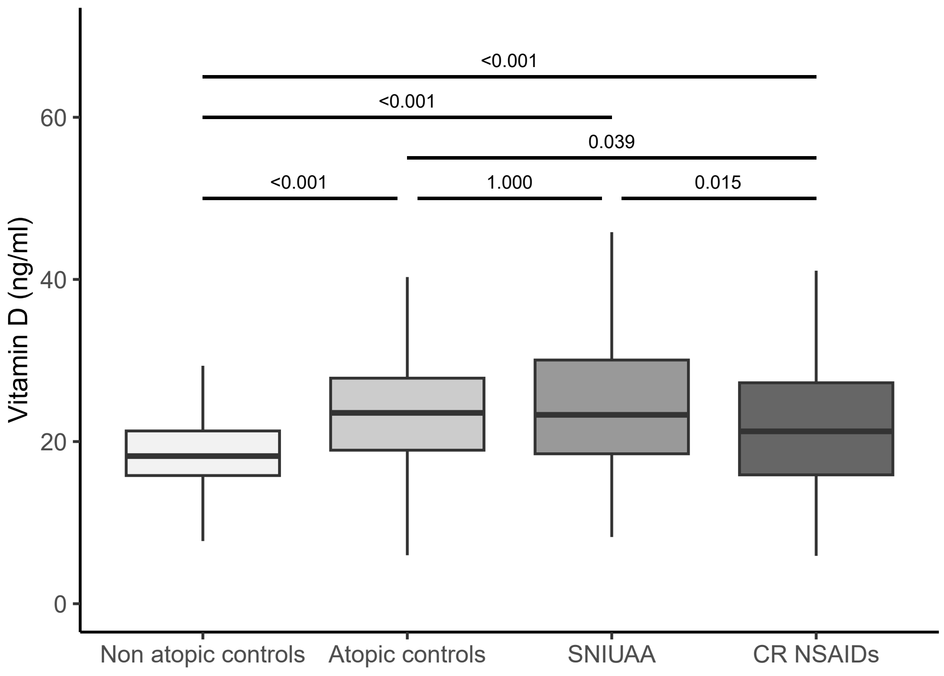

Figure S1. Box-plots of serum vitamin D levels by study group (pairwise comparison p-values obtained from Dunn's test with Bonferroni correction).
